# Comparative analysis of *cytokinin response factors* in *Brassica* diploids and amphidiploids and insights into the evolution of *Brassica* species

**DOI:** 10.1186/s12864-018-5114-y

**Published:** 2018-10-03

**Authors:** Lijun Kong, Kun Zhao, Yingying Gao, Liming Miao, Chaoquan Chen, Hang Deng, Zhenning Liu, Xiaolin Yu

**Affiliations:** 1 Laboratory of Cell and Molecular Biology, Institute of Vegetable Science, Zhejiang University, 866 Yuhangtang Road, Hangzhou, 310058 Zhejiang China; 2 Key Laboratory of Horticultural Plant Growth, Development, and Quality Improvement, Ministry of Agriculture, Zhejiang Provincial Key Laboratory of Horticultural Plant Integrative Biology, Hangzhou, 310058 Zhejiang China; 3 College of Agriculture and Forestry Sciences, Linyi University, Linyi, 276000 Shandong China

**Keywords:** *Cytokinin response factors*, *Brassica*, Comparative genomics, Evolution, Functional analysis

## Abstract

**Background:**

Cytokinin is a classical phytohormone that plays important roles in numerous plant growth and development processes. In plants, cytokinin signals are transduced by a two-component system, which involves many genes, including *cytokinin response factors* (*CRF*s). Although *CRFs* take vital part in the growth of *Arabidopsis thaliana* and *Solanum lycopersicum*, little information of the *CRFs* in the *Brassica* U-triangle species has been known yet.

**Results:**

We identified and compared 141 *CRFs* in the diploids and amphidiploids of *Brassica* species, including *B. rapa*, *B. oleracea*, *B. nigra*, *B. napus*, and *B. juncea*. For all the 141 *CRFs*, the sequence and structure analysis, physiological and biochemical characteristics analysis were performed. Meanwhile, the *Ka/Ks* ratios of orthologous and paralogous gene pairs were calculated, which indicated the natural selective pressure upon the overall length or a certain part of the *CRFs*. The expression profiles of *CRFs* in different tissues and under various stresses were analyzed in *B. oleracea*, *B. nigra*, and *B. napus*. The similarities and differences in gene sequences and expression profiles among the homologous genes of these species were discussed. In addition, *AtCRF11* and its ortholog *BrCRF11a* were identified to be related to primary root growth in Arabidopsis.

**Conclusion:**

This study performed a genome-wide comparative analysis of the *CRFs* in the diploids and amphidiploids of the *Brassica* U-triangle species. Many similarities and differences in gene sequences and expression profiles existed among the *CRF* homologous genes of these species. In the bioinformatics analysis, we found the close relativity of the *CRF* homologous genes in the *Brassica* A and C genomes and the distinctiveness of those in the B genome, and the *CRF* homologous genes in B subgenome were considerably influenced by the A subgenome of *B. juncea*. In addition, we identified a new function of the Clade V *CRFs* related to root growth, which also clarified the functional conservation between *Arabidopsis* and *B. rapa*. These results not only offer useful information on the functional analysis of *CRFs* but also provide new insights into the evolution of *Brassica* species.

**Electronic supplementary material:**

The online version of this article (10.1186/s12864-018-5114-y) contains supplementary material, which is available to authorized users.

## Background

Cytokinin is a major phytohormone that plays a key role in plant growth and development processes, such as leaf senescence [1, 2], root and shoot development [1, 3], and chloroplast development [4], as well as in biotic and abiotic stress responses [5–7]. In plants, cytokinin is perceived and responded through a multistep phosphorelay pathway, which is similar to the two-component system in bacteria [8, 9]. In *Arabidopsis thaliana*, cytokinin is recognized by sensor histidine kinases, which autophosphorylate the conserved His residue located at the kinase domain. Then, His-containing phosphotransfer proteins (HPs) transduce a signal. After a multistep His→Asp→His→Asp phosphorelay, the signal is transferred to *Arabidopsis* response regulators (ARRs), which comprise type-A ARRs, type-B ARRs, and type-C ARRs [9, 10]. Type-A ARRs are rapidly upregulated by cytokinin treatment, whereas type-B ARRs contain transactivating domains that regulate the transcription of cytokinin-activated targets, such as type-A *ARRs* [11, 12]. Cytokinin response factors (CRFs), a subset of AP2 transcription factors (TFs), are newly identified components in the cytokinin signaling pathway. They likely operate downstream of the HPs and include common or specific targets with type-B ARRs [13].

Thus far, 12 *CRFs* have been identified in *A. thaliana* [13, 14], 11 *CRFs* in *Solanum lycopersicum* [15], and 21 *CRFs* in *Brassica rapa* [16]*.* As components involved in cytokinin signal transduction, numbers of *CRFs* are closely related to plant development and stress responses [17]. For example, *CRF6* negatively regulates leaf senescence [18], whereas the overexpression lines of *CRF1*, *CRF3*, or *CRF5* display leaf senescence in early stages in *A. thaliana* [19]. *AtCRF3* and *AtCRF5* can promote the growth of lateral roots [19]. *CRFs*, such as *AtCRF2*, *AtCRF3*, and *AtCRF6*, affect female reproductive organ development by interfering with the development of the placenta and ovules [20]. In addition, numbers of CRF genes in several species are related to abiotic stress responses. In *A. thaliana*, *AtCRF4* can be induced by cold stress [21], and *AtCRF6* is related to oxidative stress and salt stress [18, 22]. In *S. lycopersicum*, *SlCRF1* can be induced by cold and drought stresses, *SlCRF2* and *SlCRF3* are involved in drought and oxidative stresses, and *SlCRF5* is related to flooding, drought, oxidative, and cold stresses [23, 24]. In *B. rapa*, *BrCRF1*, *BrCRF2*, and *BrCRF19* are upregulated by drought stress, whereas *BrCRF5* and *BrCRF21* are induced by salt stress [16]. Notably, *AtCRF2* can positively regulate salicylic acid-mediated plant immunity in *A. thaliana*, and plants overexpressing *AtCRF5* exhibit pathogen resistance. All these phenomena suggest that *CRFs* play a role in biotic stress responses [25, 26].

*Brassica* consists of numerous species with a remarkable morphological diversity [27], which is a result of the long-term evolution. There are three diploids, namely, *B. rapa* (AA, 2n = 20), *B. nigra* (BB, 2n = 16), and *B. oleracea* (CC, 2n = 18). As a result of natrual hybridization between the diploids, there are three amphidiploids, namely, *B. napus* (AACC, 2n = 38), *B. juncea* (AABB, 2n = 36), and *B. carinata* (BBCC, 2n = 34). The relationship between these six species have been illustrated through the *Brassica* U-triangle [28]. This group of species provides an excellent model for studies on species evolution by homologous recombination and polyploidization. The relationship among *Brassica* species has been characterized by many methods [29], such as genomic in situ hybridization (GISH) with fluorescence in situ hybridization (FISH) [30, 31], ribosomal DNA probes [32, 33], sequence-characterized amplified region markers [34] or repetitive sequence elements [35, 36], and simple-sequence repeat (SSR) markers [37, 38]. Meanwhile, relevant analysis of certain genes or gene families were also performed in order to elucidate the evolution of *Brassica* species [39]. Many genomes of the *Brassica* species have been sequenced [40–43], thereby providing a basis for evolutionary analysis and smoothening the process of functional gene mining.

In this study, we identified all of the CRF genes in *B. rapa*, *B. nigra*, *B. oleracea*, *B. napus*, and *B. juncea* and analyzed their phylogeny, sequence properties, and selective pressure to reveal the evolution among these species. We also examined the expression patterns of these genes in different tissues and organs or under various treatments and obtained some useful information for future functional analysis of *CRFs*. The functions of *CRFs* are substantially determined by the similarities and differences among their sequences [44, 45]. The similarities and differences among the sequences of *CRFs* in these species also provided some clues of their functions. This research not only analyzed the functions of *CRFs* but also offered insights into the evolution of *Brassica* species, which would make significant impact on functional genomics and breeding improvement in *Brassica* crops.

## Methods

### Identification of *CRFs* in *Brassica* diploid species and their amphidiploids

The protein sequences of known *CRF*s in *A. thaliana* were downloaded from TAIR and then used as seed sequences to search NCBI (https://www.ncbi.nlm.nih.gov/) for *B. rapa*, *B. oleracea*, and *B. napus* and the *Brassica* Database [46] for *B. rapa*, *B. oleracea*, *B. nigra*, *B. juncea*, and *B. napus*. BlastP search with an expected value of 100 was applied. The protein sequences of the identified *CRFs* were examined with SMART (http://smart.embl-heidelberg.de/smart/ set_mode.cgi?GENOMIC = 1) [47] and ClustalX [48]. Protein sequences that did not contain the AP2 domain or CRF domain were excluded from the further analysis. These protein sequences, CDS, and genome sequences of the *CRFs* were downloaded from the *Brassica* Database [46].

### Nomenclature and characterization analysis of *CRFs*

We identified orthologous genes by phylogenetic approach, and did blast to confirm their relations. All of the newly identified *CRF*s and *BrCRFs* were named on the basis of their orthologous genes in *A. thaliana* [14, 16], and the paralogous genes in each species were distinguished by English letters. *CRF*-related information, such as locus, chromosome position, ORF length, and deduced polypeptide length, was searched from *B. rapa* version 1.5 genome [42], *B. oleracea* version 1.1 genome [41], *B. napus* version 4.1 genome [40], *B. nigra* version 1.1 genome, and *B. juncea* version 1.5 genome [43]. The isoelectric point (pI) and molecular weight of the *CRFs* were predicted using Compute pI/Mw (http://web.expasy.org/compute_pi/) [49–51].

### Motif recognition, gene structure, and phylogenetic analysis

The motifs of *CRF*s were identified with MEME (http://meme-suite.org/tools/meme) [52]. The protein sequences of the identified *CRFs* were used for multiple-sequence alignment by ClustalW [53] with a gap open penalty of 10 and a gap extension penalty of 0.2, and unrooted phylogenetic trees were generated with the neighbor-joining method with a 1000-replicate bootstrap and other default parameters in MEGA version 6 [54]. The sequence logoes were created by WedLogo 3 (http://weblogo.threeplusone.com/). Finally, the phylogenetic tree of all *CRFs* was decorated in Itol (http://itol.embl.de/) [55]. The gene structures of *CRFs* were analyzed and drawn with Gene Structure Display Server (http://gsds.cbi.pku.edu.cn/) [56].

### Chromosome mapping and synteny analysis

Chromosomal location maps were drawn with MapChart in accordance with the positions of initiation codons and decorated in PhotoShop CS5 for the transcriptional orientation marks and bars. Synteny analysis was performed on GEvo (https://genomevolution.org/CoGe/GEvo.pl) [57] by using 100 kb sequence before and after each gene respectively, and non-CDS sequences were masked. Figures were decorated in PhotoShop CS5 to mark the gene names.

### Analysis of evolutionary selection pressure

The synonymous (*Ks*) and nonsynonymous substitution (*Ka*) rates of orthologous and paralogous genes were calculated with MEGA version 6 [54] by using a Compute Pairway Distance plate. *Ka/Ks* ratios were calculated and subtotaled in Microsoft Excel 2007, and box plots were drawn in E Chart (http://www.ehbio.com/ImageGP/index.php/Home/Index/ Boxplot.html). *Ka/Ks* values of orthologous genes between *Brassica* species and *A. thaliana* with a sliding window of 20 codons were calculated and drafted in MATLAB R2017b [58].

### Analysis of the putative promoter regions of *CRFs*

The upstream sequences (1500 bp) of the initiation codons of *BrCRFs*, *BolCRFs*, *BniCRFs*, *BnaCRFs*, and *BjuCRFs* were chosen as the putative promoter regions of *CRFs* and used to identify the *cis*-elements related to hormones and stresses. The *cis*-regulatory elements along the putative promoter sequences were identified by using PLACE (https://sogo.dna.affrc.go.jp/cgi-bin/sogo.cgi?lang=en&pj=640&action=page&page=newplace) [59] and PlantCARE (http://bioinformatics.psb.ugent.be/webtools/plantcare/html/) [60].

### Plant growth and treatments

*B. oleracea* cv. Sanxiong was grown in the experimental farm of the Zhejiang Academy of Agricultural Sciences, whereas *B. napus* line 166–13 and *B. nigra* line 1611–01 were cultivated in the experimental farm of Zhejiang University. Roots, floral stems, leaves, flowers, siliques, sepals, petals, stamens, and pistils were sampled to analyze tissue- and organ-specific expression.

The three materials were also cultivated under a 14 h light/10 h dark photoperiod at 24 °C/22 °C for about 3 weeks before treatments. For the exogenous hormone treatments, 100 μM 6-BA, NAA, and abscisic acid (ABA) were sprayed onto the three materials, and controls were sprayed with double distilled water only. All of the materials were sampled at 0, 0.5, and 1 h after treatment. In the salt treatment, a nutrient solution with 200 mM NaCl was used as a treatment, and a normal nutrient solution was utilized as control. The materials were sampled at 0, 4, 8, and 16 h after treatment. The second true leaves in all of the treatment groups were sampled to minimize differences. All of the samples were frozen in liquid nitrogen immediately and then stored at − 75 °C.

### RNA extraction and qRT-PCR analysis

For *B. napus* and *B. nigra* samples, TRIzol reagent (Invitrogen, Germany) was used to extract total RNA in accordance with the manufacturer’s instructions, and the first cDNA strand was synthesized using a TaKaRa reverse transcription system (Japan) in accordance with the manufacturer’s protocol. For *B. oleracea* samples, RNA extraction kits (Omega, USA) were utilized in accordance with the manufacturer’s instructions. qRT-PCR was performed with Primers for qRT-PCR (Additional file 1: Table S1) were designed by Primer version 5.0. A qRT-PCR mixture was 15 μL in volume and composed of 7.5 μL of SYBR Green Master Mix reagent (Toyobo, Japan), 0.6 μL of specific primer, 2 μL of cDNA, and 5.9 μL of ddH_2_O. qRT-PCR was run using a StepOne real-time PCR machine (BioRAD, USA) programmed to heat for 30 s at 95 °C, followed by 40 cycles of 5 s at 95 °C and 45 s at 55 °C–58 °C. The specificity of the reactions was verified through melting curve analysis. *GAPDH* [61] and *25S* [62] were used as internal controls. Comparative ΔΔ^CT^ method was applied to analyze the relative expression levels of *CRFs*. Hierarchical clustering and heatmap representation of the expression pattern of *CRFs* were drawn in HemI [63]. Three biological replicates were included for each sample.

### *Arabidopsis* mutant and transformation

*Atcrf11* (AT3G25890) mutant was purchased from ABRC (SALK205786C). The full-length coding sequence of *BrCRF11a* was inserted into the pCAMBIA1301 vector and driven by the CaMV 35S promoter. The constructed vector was transformed into *Arabidopsis* plants by the floral-dip method with *Agrobacterium* strain GV3101 to obtain *BrCRF11a-*overexpressing transgenic *Arabidopsis*. The relative expressions of *BrCRF11a* and *AtCRF11* in the three types of *Arabidopsis* seedlings were analyzed by qRT-PCR. *AtTUB4* (*TUBULIN BETA CHAIN 4*, AT5G44340) was used as internal reference. The primers used here can be found in the Additional file 1: Table S1. All of the *Arabidopsis* plants (wild-type *Col-0*, *Atcrf11* mutant and *BrCRF11a-*overexpressing transgenic *Arabidopsis p35S::BrCRF11a*) were grown on Murashige and Skoog (MS) medium for 5 days before the root lengths of the seedlings were measured.

## Results

### Identification, classification, and phylogenetic analysis of *CRF*s in the *Brassica* genomes

A total of 120 new *CRFs*, which simultaneously contain the AP2 domain and the CRF domain, were identified in *Brassica* species: 18 *CRFs* in *B. oleracea*, 24 *CRFs* in *B. nigra*, 38 *CRFs* in *B. juncea*, and 40 *CRFs* in *B. napus* (Table 1). The results searched from *B. rapa* version 1.5 genome were consistent with those from former research [16]. All of the 120 genes were named, and the 21 *CRFs* in *B. rapa* were renamed on the basis of their orthologs in *A. thaliana*. The paralogs were distinguished by English letters (Additional file 2: Table S2). In addition, the basic information of the genes was searched. The length of the gene sequences ranged from 441 bp to 1151 bp. In all of the species, the shortest gene sequence was that of *CRF7*, and the longest gene sequence was that of *CRF3*, except *B. juncea* in which the longest was *CRF12*. For the protein length (147–364 amino acids) and molecular weight (16.1–41.4 kDa), the shortest was found in CRF7, and the longest was observed in CRF3 in all of the species herein. The pI of the proteins ranged from 4.56 to 10.00. The protein with the lowest pI was detected in CRF3 in all of the species except *B. juncea*, whose CRF10 was the protein with the lowest pI. The protein with the highest pI was identified in CRF7 in all of the species except *B. nigra*, whose CRF8 was the protein with the highest pI.

**Table 1:** Numbers of *CRFs* in *Brassica* species

Gene		CRF1	CRF2	CRF3	CRF4	CRF5	CRF6	CRF7	CRF8	CRF9	CRF10	CRF11	CRF12	Total
Species														
*A. thaliana*		1	1	1	1	1	1	1	1	1	1	1	1	12
*B. rapa*		1	2	3	3	1	1	2	2	0	3	2	1	21
*B. oleracea*		1	1	3	3	1	2	2	2	0	2	1	0	18
*B. nigra*		1	2	3	3	2	3	2	2	0	4	2	0	24
*B. juncea*	A^a^	1	2	3	0	2	2	2	1	0	3	2	1	19
	B^a^	1	1	2	0	0	2	1	3	0	2	1	1	14
	U^a^	0	1	1	1	1	0	0	0	0	1	0	0	5
*B. napus*	A^a^	1	2	2	3	1	2	2	2	0	4	1	0	20
	C^a^	1	2	3	3	1	2	2	2	0	2	1	1	20
Total		8	14	21	17	10	15	14	15	1	22	11	5	153

^a^means subgenome of the allotetraploid; “U” means unknown

**Fig. 1:**
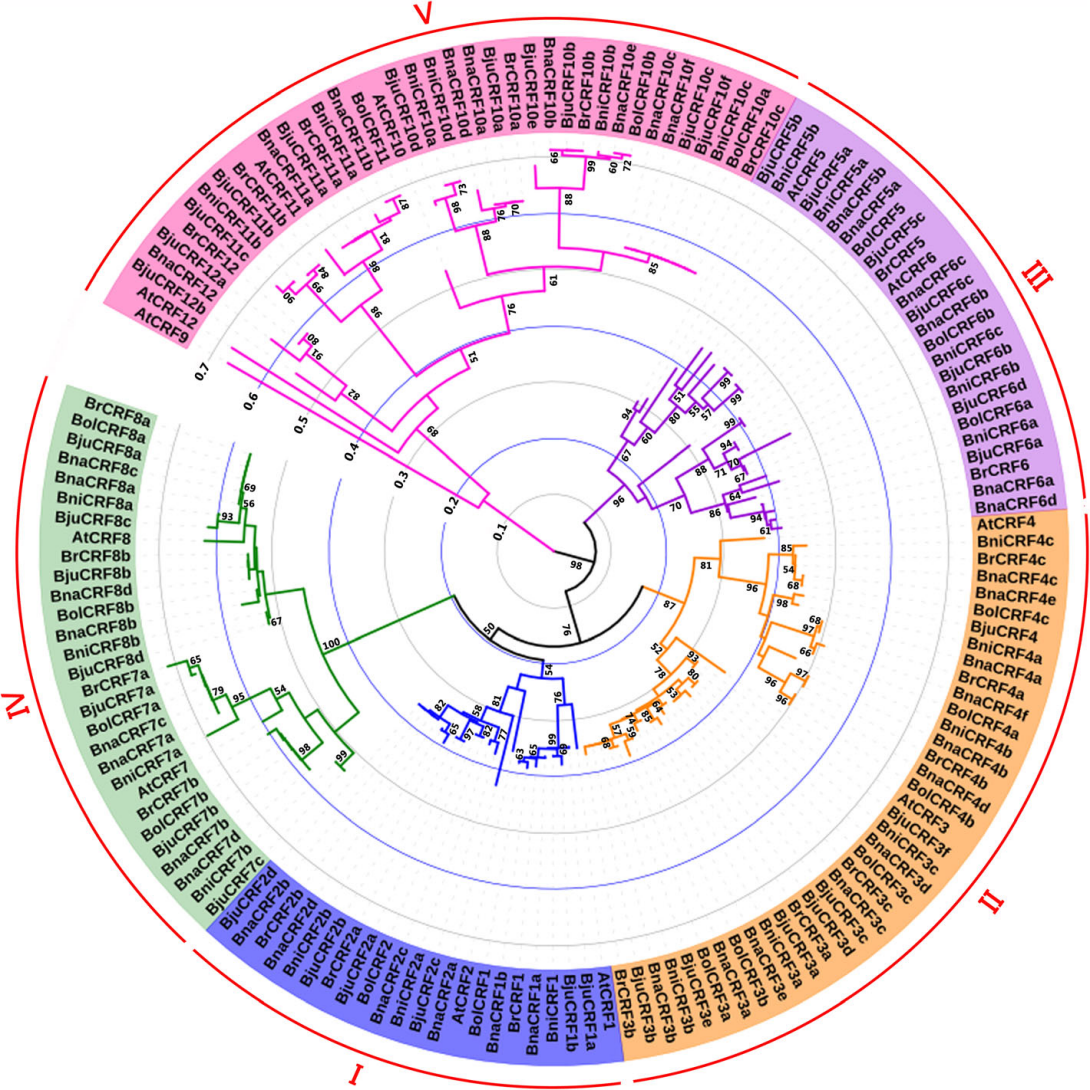
Phylogenetic tree based on CRF homologous protein sequences in *Brassica* plants. The CRFs are divided into five clades and marked by different colors. The equidistant rings indicate the scale of relative divergence. The bootstrap values were calculated from 1000 replicates and the values less than 50% were hided. The figure was obtained by MEGA version 6 and Itol (http://itol.embl.de/)

A phylogenetic tree was constructed using the protein sequences of the genes (Fig. 1). This result showed that the orthologs among genomes and the paralogs in one species were closely related. The genes can be divided into five clades: Clade I (*CRF1s* and *CRF2s*), Clade II (*CRF3s* and *CRF4s*), Clade III (*CRF5s* and *CRF6s*), Clade IV (*CRF7s* and *CRF8s*), and Clade V (*CRF9–12 s*). We found that Clades I, II, III, and V were closely related with one another, whereas Clade IV was distantly related with them.

### Gene structure and conserved motif analysis of *CRFs* in *Brassica* species

**Fig. 2:**
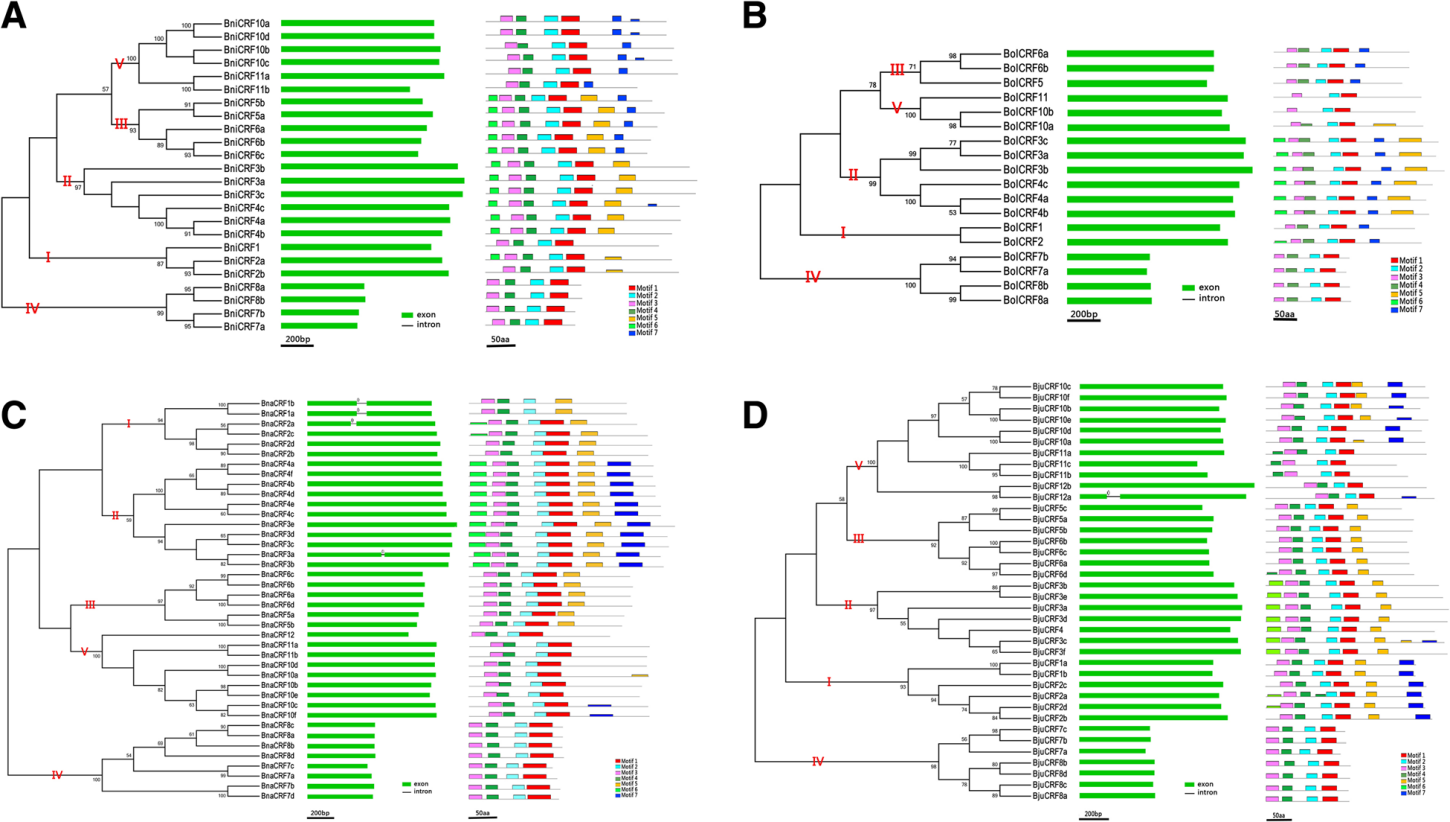
Phylogenetic relationships, gene structures, and motif analysis of *CRFs* in *Brassica* species. The *CRFs* in *B. nigra* (**a**), *B. oleracea* (**b**), *B. napus* (**c**), and *B. juncea* (**d**) are shown. In every figure, the phylogenetic relationship is shown on the left, the gene structure in middle, and the motifs on the right. The genes belonging to different clades were marked by red Latin numbers. Motif 1 and Motif 2 formed the AP2 domain. The CRF domain was comprised of Motif 3 and Motif 4. Motif 5 was a putative mitogen-activated protein kinase (MAPK) phosphorylation site. Motif 6 represented the TEH region

The phylogenetic trees of *CRFs* in *B. nigra* (Fig. 2a), *B. oleracea* (Fig. 2b), *B. napus* (Fig. 2c), and *B. juncea* (Fig. 2d) were constructed separately. Five clades remained in each species, and this finding was consistent with the previous phylogenetic analysis (Fig. 1). Notably, all of the species here did not possess any other orthologs of *AtCRF9*. Gene structure analysis showed that most *CRFs* had only one exon except *BnaCRF1a*, *BnaCRF1b*, *BnaCRF2a*, *BnaCRF3a*, and *BjuCRF12a*, which contained two exons and one intron (Fig. 2). Conserved motif analysis and multiple alignment were performed using the *CRFs*’ protein sequences. The sequences could be divided into three types based on sequence similarity: Type A included proteins in Clades I, II, and III (CRF1–6 s), Type B comprised the proteins in Clade IV (CRF7s and CRF8s), and Type C consisted of proteins in Clade V (CRF9–12 s). All of the proteins had one AP2 domain (Motif 1, 2) and one CRF domain (Motif 3, 4). The TEH region (Motif 6) only existed in the N-terminal region of Type A proteins, and all of the Type A proteins contained a putative mitogen-activated protein kinase (MAPK) phosphorylation site (Motif 5) on the C-terminal region. Type B proteins were shorter and did not have motifs other than the AP2 and CRF domains. The length of the Type C proteins was similar to that of the Type A proteins. However, the former did not contain a TEH region and an MAPK motif. Instead, they possessed motifs with consensus sequences [FNF × ×L × IPD] and [GPSxLPD×DF × DV] (Fig. 2 and Additional file 3: Figure S1).

### Chromosomal distribution comparison and synteny analysis of *CRFs* among *Brassica* species

A refined analysis of synteny blocks was performed by GEvo website (Additional file 4: Figure S2) and provided some clear hints to the origin of genes in allotetraploids (Table 2 and Additional file 5: Table S3). The gene origins of most of the genes in the allotetraploids could be found in the diploids, whereas few of them were newly generated. Moreover, some genes in the diploids did not have corresponding genes in the allotetraploids. In addition, some genes (*BnaCRF2d*, *BnaCRF6c*, *BjuCRF6c and BjuCRF12b*) were highly similar to their paralogous genes (Additional file 4: Figure S2).

**Table 2:** List of origin genes in *B. oleracea* and amphidiploid genes in *B. napus*

Origin gene in *B. oleracea*	Amphidiploid gene in *B. napus*
*BolCRF1*	*BnaCRF1b*
*BolCRF2*	*BnaCRF2c*
*BolCRF3a*	*BnaCRF3a*
*BolCRF3c*	*BnaCRF3d*
*BolCRF4a*	*BnaCRF4f*
*BolCRF4b*	*BnaCRF4d*
*BolCRF5*	*BnaCRF5a*
*BolCRF6a*	*BnaCRF6d*
*BolCRF6b*	***BnaCRF6b*** ***BnaCRF6c***
*BolCRF7a*	*BnaCRF7a*
*BolCRF7b*	*BnaCRF7b*
*BolCRF8a*	*BnaCRF8c*
*BolCRF8b*	*BnaCRF8b*
*BolCRF10a*	*BnaCRF10f*
*BolCRF10b*	*BnaCRF10e*
*BolCRF11*	*BnaCRF11b*

High-similarity genes are marked in bold

**Fig. 3:**
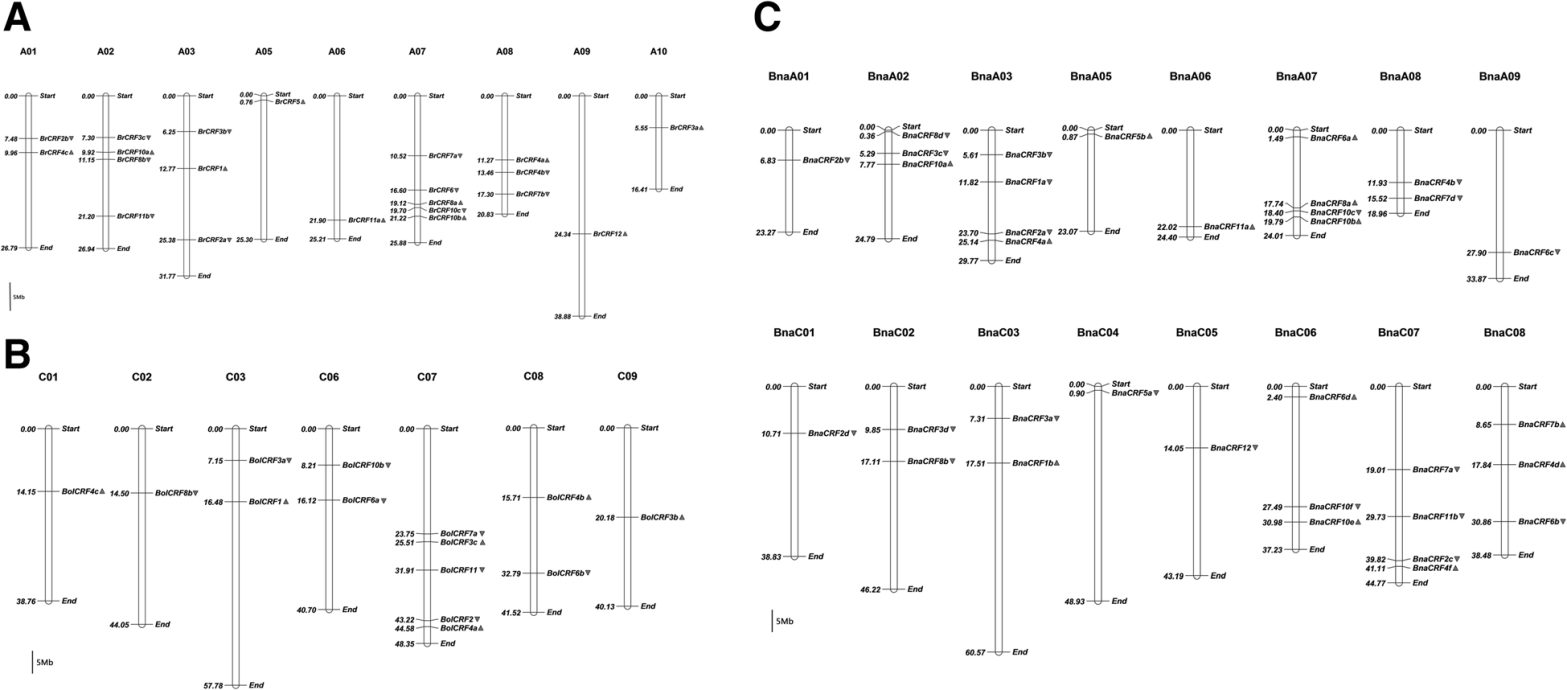
Chromosomal mapping of *CRFs* in *B. rapa*, *B. oleracea*, and *B. napus*.. The *CRFs* in *B. rapa* (**a**), *B. oleracea* (**b**), and *B. napus* (**c**) are shown except those located on the scaffolds. The locations were shown on the left of the chromosomes, whereas the gene names were on the right. The arrows next to gene names show the direction of transcription. The bar indicates the size of 5 Mb

Chromosomal mapping of *CRFs* in *B. rapa*, *B. oleracea*, *B. nigra*, *B. juncea*, and *B. napus* was performed separately on basis of genomic information (Fig. 3 and Additional file 6: Figure S3). Results showed that the *CRFs* were distributed throughout almost all the chromosomes in every genome, except A04 in *B. rapa*; B05 in *B. nigra*; C04 and C05 in *B. oleracea*; A04, A10, and C09 in *B. napus*; and A04 and B01 in *B. juncea.*

By comparing the *B. oleracea* genome with the C subgenome of *B. napus* (Fig. 3b and c), the chromosomal location of most allotetraploid genes are similar to their origin genes. Although some genes, such as *BolCRF5*, *BolCRF7a*, *BolCRF8a*, *BolCRF10a*, and *BnaCRF8c*, possess incomplete location information, their corresponding genes offer hints. However, gene rearrangements are also present. For example, *BolCRF3c* is located in chromosome C07 in *B. oleracea*, and its corresponding gene *BnaCRF3d* is in chromosome C02 in *B. napus*. Moreover, no origin gene of *BnaCRF2d* is present on the corresponding site in the *B. oleracea* genome. On one hand, the origin gene may have degenerated after hybridization. On the other hand, the chromosomal sequence around *BnaCRF2d* is highly similar to that of *BnaCRF2b* and may be a copy of *BnaCRF2b* derived from segmental duplication or another mechanism. Indeed, *BolCRF3b* and *BolCRF4c* possess no corresponding gene in the other genome. The relationship of the corresponding genes between *B. rapa* and the A subgenomes of *B. napus* and *B. juncea* is similar to that between *B. oleracea* and the C subgenome of *B. napus* (Fig. 3a, c and Additional file 6: Figure S3B). In the A subgenome of *B. napus*, *BnaCRF4a*, *BnaCRF6a*, and *BnaCRF8d* are rearranged after hybridization, and the corresponding genes of *BrCRF3a*, *BrCRF4c*, *BrCRF7a*, and *BrCRF11b* were not found. Especially, *BnaCRF12*, the corresponding gene of *BrCRF12*, is located in chromosome C05, which belongs to the C subgenome of *B. napus*. Similarly, *BnaCRF6c* is also significantly similar to *BnaCRF6b* in chromosome C08. In the A subgenome of *B. juncea*, only the corresponding genes of *BrCRF4a*, *BrCRF4b*, and *BrCRF4c* were not observed. Although the information was incomplete for *BjuCRF4*, the number of CRF4 genes decreased significantly in *B. juncea*. By contrast, no origin gene of *BjuCRF5b* was found in *B. rapa*; the gene possibly degenerated after hybridization. *BjuCRF8a*, the corresponding gene of *BrCRF8a*, was rearranged from the A subgenome to C subgenome. At the same time, *BjuCRF6c* was highly similar to *BjuCRF6b*, which was coincident with *BnaCRF6c* in *B. napus*. This situation was also noted between *BjuCRF12a* and *BjuCRF12b*.

These findings were much more complicated for *B. nigra* and *B. juncea* (Additional file 6: Figure S3). All the corresponding genes were rearranged, and no significant regularity was found among these genes. A total of 7 of 24 *CRFs* in *B. nigra* have no corresponding genes in *B. juncea*, and this proportion was higher than that in *B. rapa* (1 of 21) and in *B. oleracea* (2 of 18).

### Gene retention ratio and evolutionary selection pressure analysis of *CRFs* in the *Brassica* species

The numbers of *CRFs* counted by genome (subgenome) and gene name separately are displayed in Table 1. Genome triplication occurred in the *Brassica* species since their divergence from the *A. thaliana* lineage at about 13–17 million years ago (MYA) [42]. Thus, the diploids and their amphidiploids reasonably possess 3 and 6 paralogs, respectively, for every corresponding CRF gene in *A. thaliana*. On this basis, gene retention ratios were calculated by genome (subgenome) and gene name separately (Additional file 7: Figure S4). Although the gene retention ratios in the genomes or subgenomes were almost the same, the gene retention ratios for *B. nigra* and *B. rapa* were higher, but lower for the B subgenome in *B. juncea*, than that in the other species. For the gene retention ratios of different genes, more *CRF10* genes and less *CRF12* genes were maintained. Furthermore, no *CRF9* gene existed in the *Brassica* species. In addition, the gene retention ratios of *CRFs* in the three sub-genomes of *B. rapa*, *B. oleracea*, and *B. napus* were analyzed (Additional file 8: Figure S5.). The result showed that the gene retention ratios of *CRFs* in the three sub-genomes were the same in *B. oleracea*. In *B. rapa*, *B. napus A*, and *B. napus C*, the gene retention ratios of *CRFs* were higher in LF and MF1 and lower in MF2. Furthermore, the gene retention ratios of *CRFs* in the three sub-genomes in *B. rapa* and *B. oleracea* were higher (0.5 or 0.58) than that in *B. napus A* and *B. napus C* (0.33 or 0.42).

**Fig. 4:**
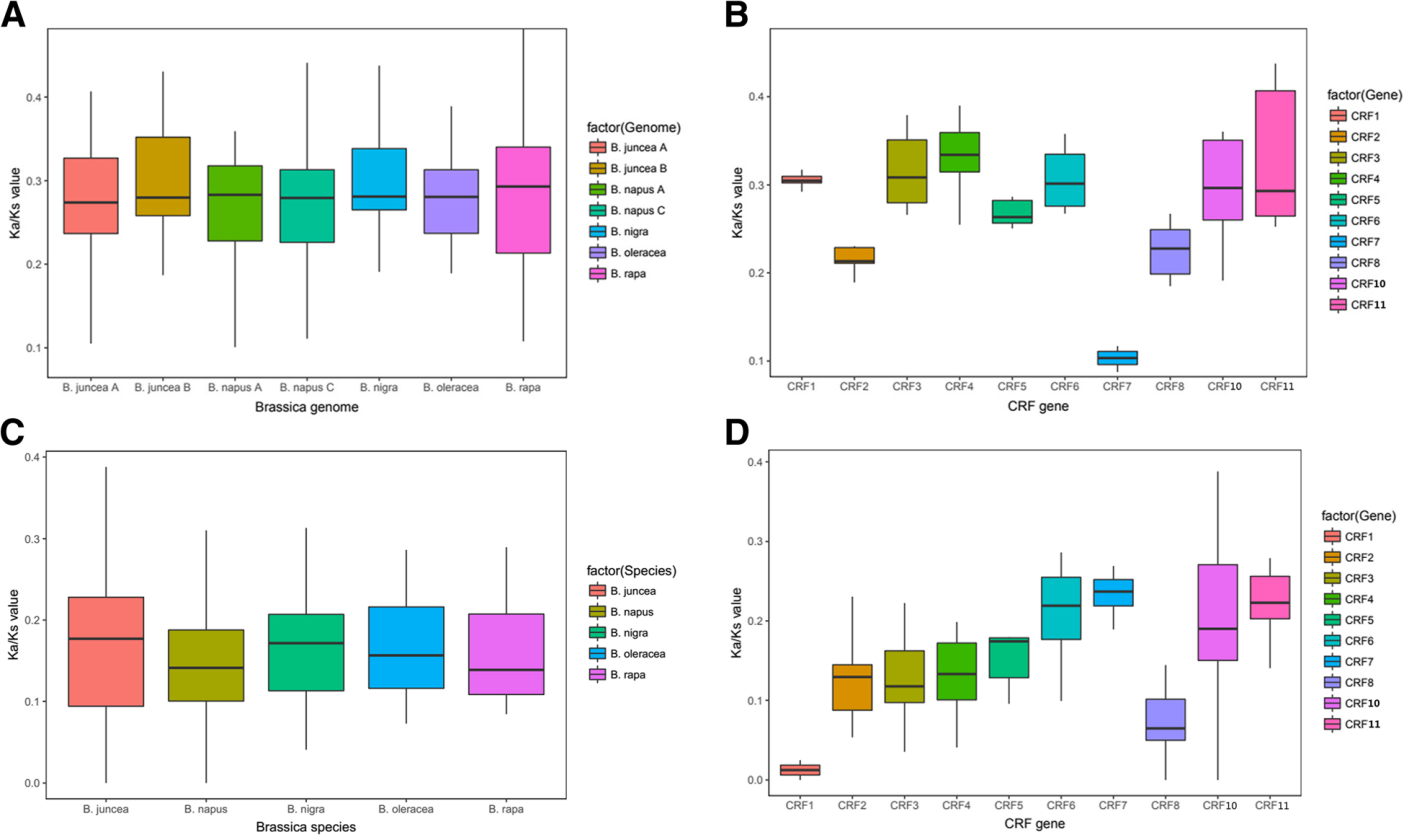
Box-plots of the *Ka/Ks* values of homologous gene pairs in *Brassica* species and *Arabidopsis thaliana*. Orthologous gene pairs between *Brassica* species and *Arabidopsis thaliana* (**a** and **b**) and paralogous gene pairs among each *Brassica* species (C and D) were shown. The statistics by species (**a** and **c**) and by gene name (**b** and **d**) are displayed. The values of CRF9 and CRF12 are not shown because of the exceedingly low numbers

The *Ks* (synonymous substitution rates) and *Ka* (non-synonymous substitution rates) were determined to explore the gene divergence after duplication. The *Ka/Ks* value indicates the selection pressure on a gene pair. *Ka/Ks* < 1 means negative selection, *Ka/Ks* = 1 means neutral selection, and *Ka/Ks* > 1 means positive selection [64]. We obtained the *Ka/Ks* ratios from duplicated *CRF* orthologous gene pairs (Fig. 4a and b) between the *Brassica* species and *A. thaliana* and duplicated *CRF* paralogous gene pairs (Fig. 4c and d) in every *Brassica* genome. All the gene pairs underwent negative selection, and the *Ka/Ks* values ranged from 0 to 0.5. In different *Brassica* species, the *Ka/Ks* values of *CRF* gene pairs seemed to be similar. The results indicate that the *Brassica* species have encountered the similar selection pressure during the processes of domestication (Fig. 4a and c). For different *CRF* genes, in the evolution between *Brassica* species and *A. thaliana*, the *Ka*/*Ks* values of *CRF7s* were the lowest, whereas those of the *CRF11s* were the highest. However, in the *Brassica* species, the *Ka*/*Ks* values of the *CRF1s* and *CRF8s* were the lower, whereas those of *CRF6s*, *CRF7s*, *CRF10s*, and *CRF11s* were relatively higher (Fig. 4b and d).

Furthermore, we calculated the *Ka/Ks* values of the *CRF* orthologous gene pairs of *Br-At*, *Bol-At*, and *Bna-At* with a sliding window of 20 codons. Thus, we observed whether the *Ka/Ks* ratios changed in different parts of the protein (Additional file 9: Figure S6). We found that the *Ka/Ks* values of different protein parts were extremely distinct. Almost every protein contained at least one part with a *Ka/Ks* value more than 1. This result indicates that the mutations in these parts underwent positive selection. However, most of these parts avoided but were close to the conserved motifs. In addition, the C-termination parts of the proteins usually possessed relatively high *Ka/Ks* values of more than 1. Meanwhile, the orthologous genes manifested a similar variation trend.

### Analysis of the putative promoter regions of *CRFs* in *Brassica* species

Gene expression variation among species is believed to be responsible for much of the phenotypic diversity [65], and many genes, with tissue-specific or stress-responsive expression patterns, are closely related to the *cis*-regulatory elements located upstream of these genes [66]. To thoroughly understand the function of the *CRF* genes and determine the presence of functional diversity among *Brassica* species, the putative promoter regions of *CRFs* in *Brassica* species were analyzed using PLACE website. The *cis*-regulatory elements related to hormones and stresses were counted, such as ARR1AT, CPBCSPOR, NTBBF1ARROLB, MYBATRD22, CBFHV and so forth. These results showed that all the *CRFs* contained several *cis*-regulatory elements related to hormones and stresses in their promoters (Additional file 10: Table S4). In particular, elements related to cytokinin, auxin, ABA, and GA were much more than other hormones. Similarly, more cold-stress elements were present than those related to other stresses. Meanwhile, we found that for many paralogous genes, there always existed a gene containing much more elements than its paralogous genes. Meanwhile, the PlantCARE website was also utilized to analyze the promoters. Although the dataset was restricted, the result coincided with the prediction of PLACE website (Additional file 11: Table S5).

### Expression pattern of *CRFs* in different tissues and organs of the *Brassica* species

**Fig. 5:**
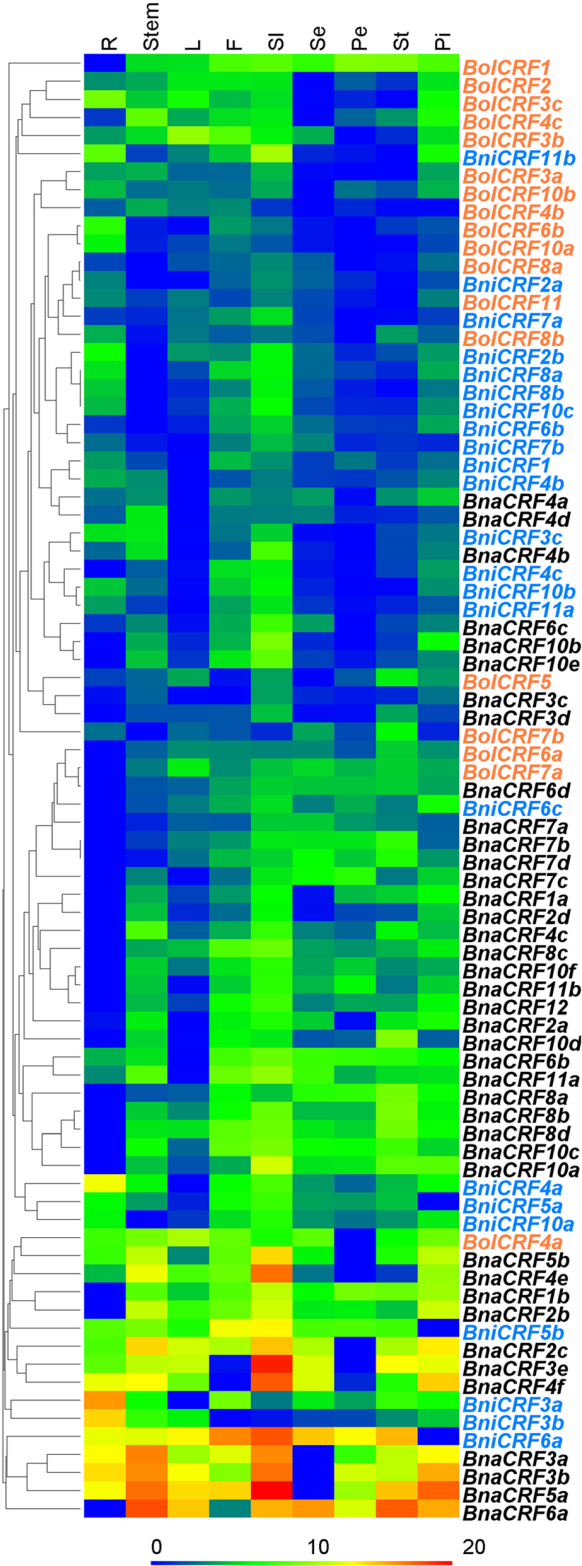
Hierarchical clustering and heatmap representation for the expression pattern of *CRFs* in different tissues and organs of *Brassica* species. *CRFs* in different species are marked by different colors: *B. oleracea* (red), *B. nigra* (blue), and *B. napus* (black). R: root, L: leaf, F: flower, Sl: silique, Se: sepal, Pe: petal, St: stamen, and Pi: pistil. The relative expression levels of genes are presented using fold-change values transformed to Log_2_ format

The temporal and spatial expression of genes is the foundation of their function [67]. To obtain some clues about the functions of *BolCRFs*, *BniCRFs*, and *BnaCRFs*, qRT-PCR was used to analyze the transcription levels of these genes in the roots, stems, leaves, flowers, and siliques (Fig. 5). This result showed that the relative expressions of 4 *BniCRFs* (*BniCRF3a*, *3b*, *5b*, and *6a*) and 11 *BnaCRFs* (*BnaCRF1b*, *2b*, *2c*, *3a*, *3b*, *3e*, *4e*, *4f*, *5a*, *5b*, and *6a*) among different tissues were much more different than those of *BolCRFs* and other *BniCRFs* or *BnaCRFs*. Meanwhile, we found that almost all the *CRFs* exhibited higher relative expression levels in flowers and siliques. A total of 42, 59, and 48 out of 81 analyzed *CRFs* also showed more transcripts in the roots, stems, and leaves, respectively. For paralogous genes in one species, some genes showed similar expression patterns in different tissues (*BolCRF3b* with *BolCRF3c*, *BniCRF8a* with *BniCRF8b*, *BnaCRF4a* with *BnaCRF4d*, and so on), while some genes expressed complementarily (*BolCRF4a* with *BolCRF4b*, *BolCRF7a* with *BolCRF7b*, *BnaCRF6a* with *BnaCRF6c*, and so on). As regards the relationship between original genes and their corresponding genes, *BolCRF1*, *BolCRF2*, *BolCRF3a*, *BolCRF3c*, *BolCRF4b*, *BolCRF5*, *BolCRF6a*, *BolCRF7a*, and *BolCRF8a* exhibited similar expression patterns to their corresponding genes, but *BolCRF4a*, *BolCRF6b*, *BolCRF7b*, *BolCRF8b*, *BolCRF10a*, *BolCRF10b*, and *BolCRF11* displayed complementary expression profiles. Furthermore, by comparing the collinearity genes between *B. oleracea* and *B. nigra*, we noted the lacking orderliness that may have been derived from the distant relationship between such species.

Because *CRFs* belong to the *AP2* super family [13] and *AP2* is considered to be important for flower development [68], we further analyzed the transcriptional levels of the *CRFs* in sepals, petals, stamens, and pistils (Fig. 5). We found that most of the *BolCRFs* and *BniCRFs* showed smaller changes of relative expression levels in the four floral organs, whereas the relative expression levels of most *BnaCRFs* changed a lot among the four floral organs. Furthermore, some genes showed pistil-preferential expression among the four floral organs. These genes were *BolCRF2*, *BolCRF3b*, *BolCRF3c*, *BolCRF4c*, *BniCRF11b*, *BnaCRF4e*, and *BnaCRF10b.* Meanwhile, *BolCRF1*, *BolCRF4a*, *BniCRF6c*, *BnaCRF1b*, *BnaCRF2b*, *BnaCRF2c*, *BnaCRF3a*, *BnaCRF3b*, *BnaCRF3e*, *BnaCRF4f*, *BnaCRF5a*, *BnaCRF5b*, and *BnaCRF6a* also revealed higher relative expression levels in pistil. On the contrary, genes such as *BolCRF7b*, *BniCRF5a*, *BniCRF5b*, *BniCRF6a*, *BnaCRF7a*, *BnaCRF7b*, and *BnaCRF7d* exhibited lower relative expression levels in the pistil but higher relative expression levels in other floral organs.

### Effects of exogenous 6-BA, NAA, and ABA on the expression of *CRFs* in *Brassica* species

**Fig. 6:**
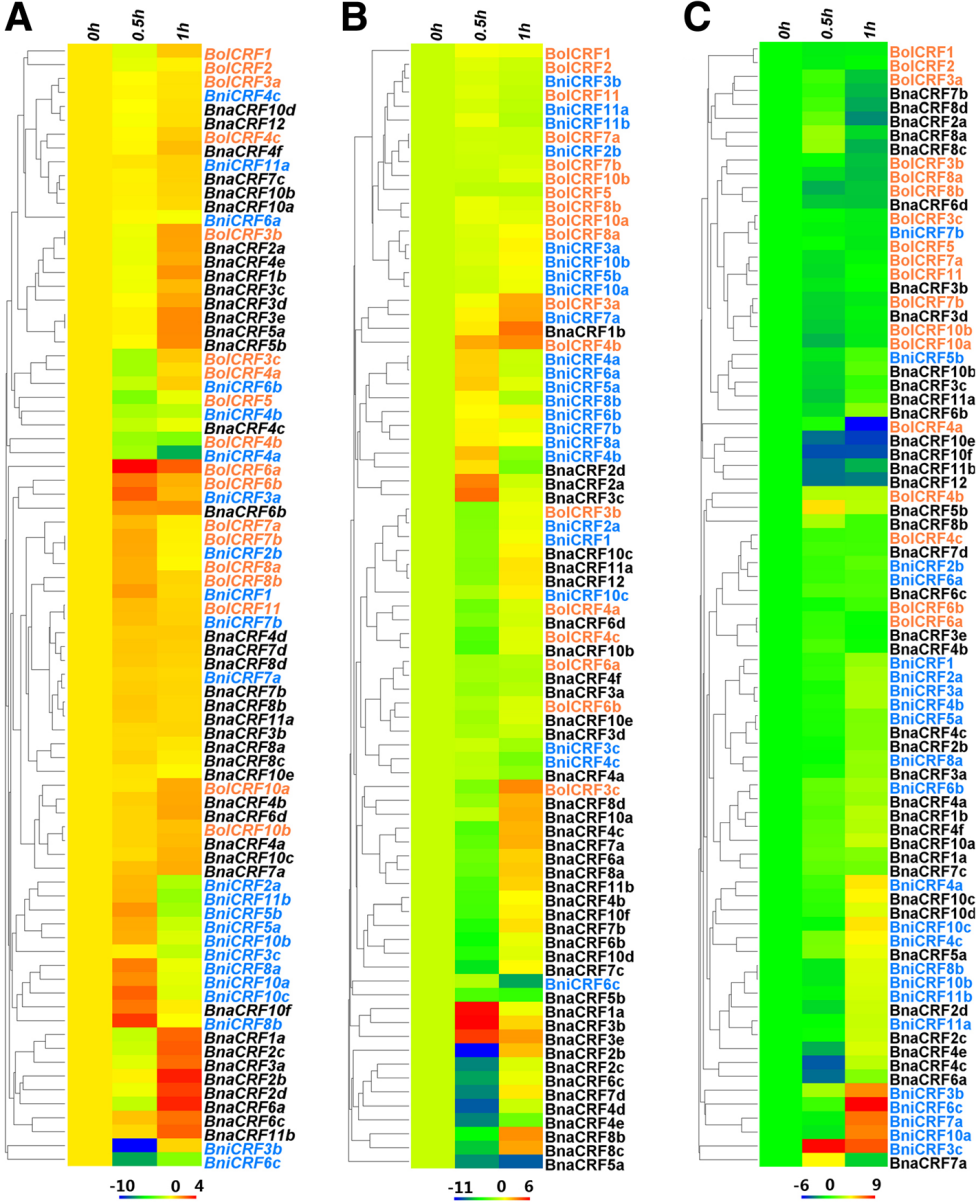
Hierarchical clustering and heatmap representation for the expression pattern of *CRFs* in *Brassica* species with exogenous 6-BA, NAA, and ABA. The genes’ relative expression levels at 0, 0.5, and 1 h after treatment with 6-BA (**a**), NAA (**b**), and ABA(**c**). The *CRFs* in different species are marked by different colors: *B. oleracea* (red), *B. nigra* (blue), and *B. napus* (black). The relative expression levels of genes are presented using fold-change values transformed to Log_2_ format

*CRF* genes were firstly discovered to respond to exogenous cytokinin, which explains their designation as *CRFs* [13]. To confirm whether the *CRFs* respond to cytokinin, qRT-PCR was applied to test the relative expression levels of the *CRFs* at 0.5 and 1 h after 6-BA treatment. Results showed most of the *CRFs* tested responded to exogenous cytokinin in various degrees (Fig. 6a). *BolCRF3b*, *BolCRF6a*, *BolCRF6b*, *BolCRF10a*, *BniCRF1*, *BniCRF2b*, *BniCRF3a*, *BniCRF8a*, *BniCRF8b*, *BniCRF10a*, *BniCRF10c*, *BnaCRF1b*, *BnaCRF2a*, *BnaCRF2b*, *BnaCRF2d*, *BnaCRF3c*, *BnaCRF3d*, *BnaCRF3e*, *BnaCRF4b*, *BnaCRF4e*, *BnaCRF5a*, *BnaCRF5b*, *BnaCRF6b*, *BnaCRF6c*, *BnaCRF6d*, *BnaCRF7a*, *BnaCRF10c*, *BnaCRF10f*, and *BnaCRF11b* were up-regulated significantly, whereas *BolCRF3c*, *BolCRF4a*, *BolCRF4b*, *BolCRF5*, *BniCRF3b*, *BniCRF3c*, *BniCRF4a*, *BniCRF4b*, *BniCRF6c*, and *BnaCRF4c* were obviously down-regulated. The genes initially up-regulated then down-regulated were *BniCRF2a*, *BniCRF5a*, *BniCRF5b*, *BniCRF10b*, and *BniCRF11b*, whereas those initially down-regulated then up-regulated were *BniCRF6b*, *BnaCRF1a*, *BnaCRF2c*, and *BnaCRF6a*. Three pairs of genes in *B. oleracea* and *B. napus* (*BolCRF6a* with *BnaCRF6d*, *BolCRF6b* with *BnaCRF6b*, and *BolCRF10a* with *BnaCRF10f*) showed the same response profiles. No significant regularity was recognized between *B. oleracea* and *B. nigra.*

In addition, more than 20 auxin-related genes can also be regulated by cytokinin, this observation suggests that auxin–cytokinin crosstalk may result from some genes co-regulated by the two hormones [69]. We analyzed the relative expression levels of the *CRFs* at 0.5 and 1 h after NAA treatment; the results revealed that numbers of genes can be regulated by NAA (Fig. 6b). On one hand, some genes were significantly up-regulated and included *BolCRF3a*, *BolCRF4b*, *BniCRF4a*, *BniCRF5a*, *BniCRF6a*, *BniCRF7a*, *BniCRF10c*, *BnaCRF1a*, *BnaCRF1b*, *BnaCRF2a*, *BnaCRF3b*, *BnaCRF3c*, *BnaCRF3e*, and *BnaCRF10a.* On the other hand, several genes were down-regulated obviously, namely, *BolCRF3b*, *BolCRF4a*, *BolCRF4c*, *BolCRF6a*, *BniCRF1*, *BniCRF2a*, *BniCRF4c*, *BniCRF6c*, *BnaCRF2c*, *BnaCRF3a*, *BnaCRF3d*, *BnaCRF4a*, *BnaCRF4d*, *BnaCRF4e*, *BnaCRF4f*, *BnaCRF5a*, *BnaCRF5b*, *BnaCRF6b*, *BnaCRF6d*, *BnaCRF10b*, *BnaCRF10d*, and *BnaCRF10e*. Meanwhile, *BolCRF3c*, *BnaCRF2b*, *BnaCRF4b*, *BnaCRF4c*, *BnaCRF6a*, *BnaCRF7a*, *BnaCRF7b*, *BnaCRF7c*, *BnaCRF7d*, *BnaCRF8a*, *BnaCRF8b*, *BnaCRF8c*, *BnaCRF8d*, *BnaCRF10c*, *BnaCRF10f*, *BnaCRF11a*, *BnaCRF11b*, and *BnaCRF12* were initially down-regulated and then up-regulated. Conversely, *BniCRF4b* and *BniCRF8b* were initially up-regulated then down-regulated. Significantly, the paralogous genes of *BnaCRF1*, *BnaCRF5*, *BnaCRF7*, *BnaCRF8*, and *BnaCRF11* displayed similar response profiles. The response profiles to NAA of two genes, namely, *BolCRF4a* and *BolCRF6a*, were similar to those of their corresponding genes in *B. napus* (*BnaCRF4f* and *BnaCRF6d*, respectively).

Analysis of the promoter regions of the *CRFs* showed the existence of several elements related to ABA. Therefore, ABA treatment was performed to test whether the *CRFs* can response to exogenous ABA. qRT-PCR was performed to analyze the relative expression levels of the *CRFs* at 0.5 and 1 h after ABA treatment. In fact, many *CRFs* responded to ABA (Fig. 6c). *BolCRF4b*, *BniCRF1*, *BniCRF2a*, *BniCRF3a*, *BniCRF3b*, *BniCRF3c*, *BniCRF4a*, *BniCRF4b*, *BniCRF4c*, *BniCRF6c*, *BniCRF7a*, *BniCRF8b*, *BniCRF10a*, *BniCRF10b*, *BniCRF10c*, *BniCRF11a*, *BniCRF11b*, *BnaCRF1b*, *BnaCRF2c*, *BnaCRF5a*, *BnaCRF5b*, *BnaCRF7a*, *BnaCRF10a*, *BnaCRF10c*, and *BnaCRF10d* were up-regulated by ABA. On the contrary, ABA can significantly down-regulate *BolCRF3a*, *BolCRF3b*, *BolCRF4a*, *BolCRF6a*, *BolCRF8a*, *BolCRF8b*, *BolCRF10a*, *BnaCRF2a*, *BnaCRF6a*, *BnaCRF6d*, *BnaCRF7b*, *BnaCRF8c*, *BnaCRF8d*, *BnaCRF10e*, *BnaCRF10f*, *BnaCRF11a*, *BnaCRF11b*, and *BnaCRF12.* Meanwhile, *BnaCRF2d*, *BnaCRF4c*, and *BnaCRF4e* are down-regulated initially and subsequently up-regulated*.* Especially, paralogous genes of *BniCRF3*, *BniCRF4*, *BniCRF10*, *BniCRF11*, *BolCRF8*, and *BnaCRF5* show similar response profiles, and the response profiles of *BolCRF6a*, *BolCRF8a*, and *BolCRF10a* are consistent with those of their corresponding genes in *B. napus* (*BnaCRF6d*, *BnaCRF8c*, and *BnaCRF10f*).

### Expression profiles of *BolCRFs*, *BniCRFs*, and *BnaCRFs* under salt stress

**Fig. 7:**
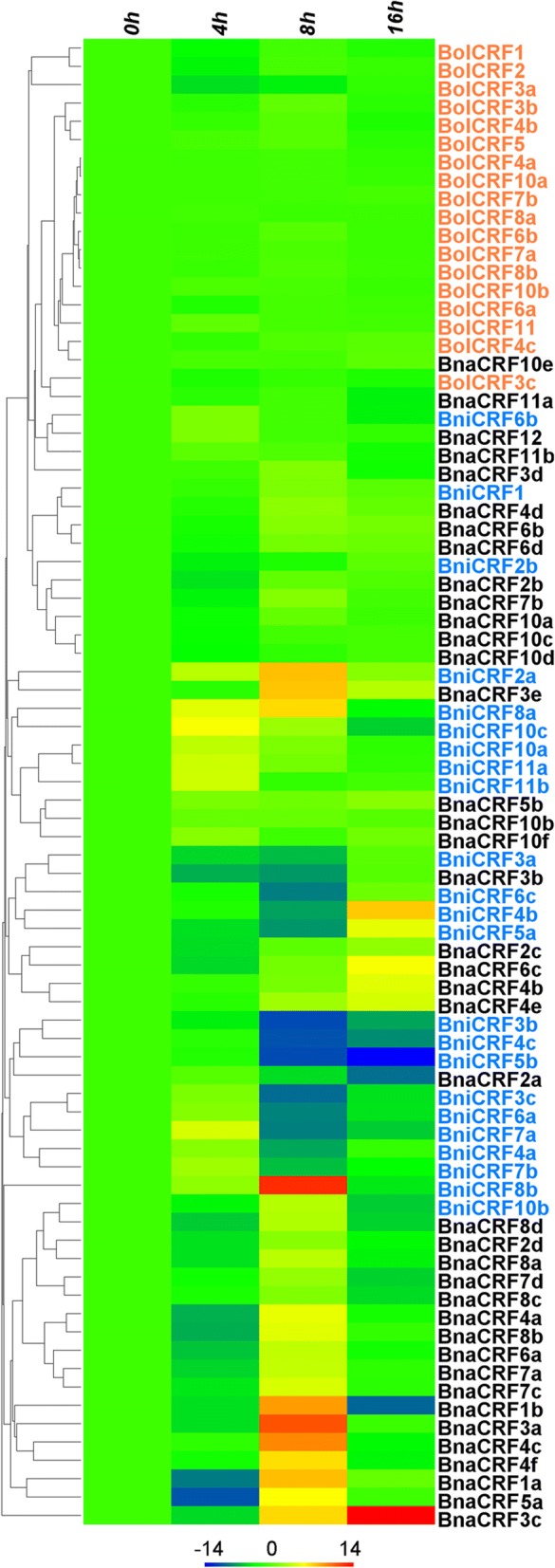
Hierarchical clustering and heatmap representation for the expression patterns of *CRFs* in *Brassica* species under salt stress. The genes’ relative expression levels at 0, 4, 8, and 16 h after salt treatment. The *CRFs* of different species are marked by different colors: *B. oleracea* (red), *B. nigra* (blue), and *B. napus* (black). The relative expression levels of genes are presented using fold-change values transformed to Log_2_ format

To date, many genes belonging to the *AP2/ERF* gene family from various plant species have been shown to be involved in abiotic stress responses [70, 71], especially in the drought and salt stress response [72]. The expression profiles of *CRFs* under salt stress for 4, 8, and 16 h were analyzed by qRT-PCR (Fig. 7). In *B. oleracea*, *BolCRF3b* and *BolCRF11* were up-regulated, while *BolCRF1*, *BolCRF2*, *BolCRF3a*, *BolCRF3c*, and *BolCRF4b* were down-regulated continuously after salt treatment. The expressions of other *BolCRFs* were not changed. In *B. nigra*, 5 genes (*BniCRF1*, *BniCRF2a*, *BniCRF10a*, *BniCRF11a*, and *BniCRF11b*) were up-regulated, while 4 genes (*BniCRF3a*, *BniCRF3b*, *BniCRF4c*, and *BniCRF5b*) were down-regulated sustainedly, and the expression changes of the other *BniCRFs* were zigzag. In *B. napus*, 8 genes were up-regulated persistently, namely *BnaCRF3e*, *BnaCRF4b*, *BnaCRF4e*, *BnaCRF5b*, *BnaCR10b*, *BnaCR10e*, *BnaCRF10f*, and *BnaCRF12*. Five genes, consist of *BnaCRF2a*, *BnaCRF3b*, *BnaCRF10c*, *BnaCRF10d*, and *BnaCRF11a*, were down-regulated continuously. However, the expression changes of the other *BnaCRFs* were fluctuant under salt stress.

### Functional analysis of *AtCRF11* and *BrCRF11a* in the root growth of *Arabidopsis*

**Fig. 8:**
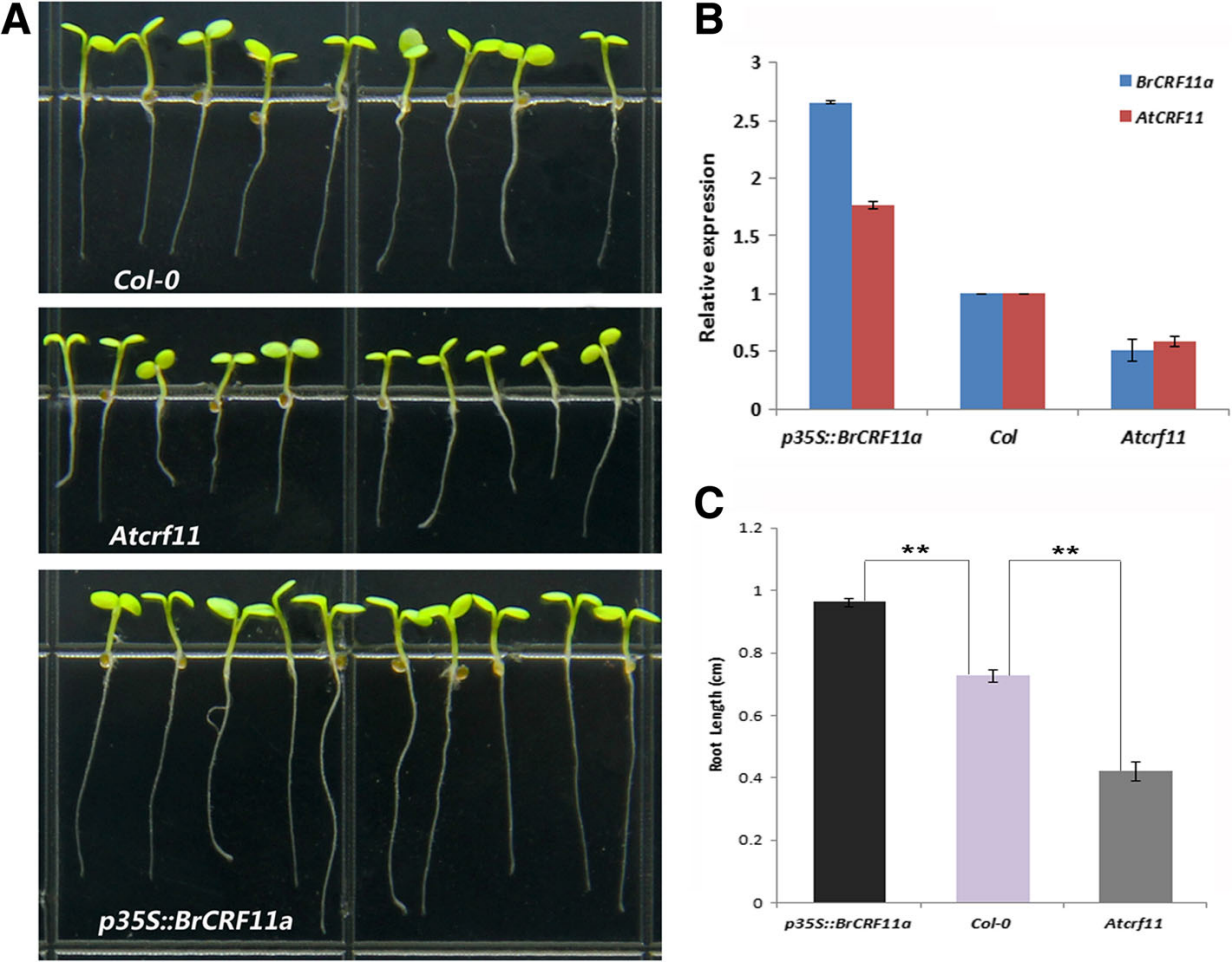
Functional analysis of *AtCRF11* and *BrCRF11a* in the root growth of *Arabidopsis*. **a** Five-day-old seedlings growing on MS medium. The length of a square slide is about 1.2 cm. **b** Relative expression analysis of *BrCRF11a* and *AtCRF11* in the three types of *Arabidopsis.* **c** Statistical analysis of the changing root length among the three types of *Arabidopsis.* “*t”* test was applied, and *P* < 0.01 is marked by “**”

Many *CRFs* were found to be highly expressed in the root, and some can even modulate root growth in *Arabidopsis* [19, 22]*.* However, most studies focused on the *CRFs* in Clades I, II, III, and IV; studies on the *CRFs* of Clade V are limited. Interestingly, in our previous study, we found that some *CRFs* in Clade V, such as *CRF11*, exhibited preferential expression in the root of *B. rapa*. To examine whether *AtCRF11* and its orthologous gene *BrCRF11a* play a role in the root growth of *Arabidopsis*, we grew three types of *Arabidopsis* (wild type *Col-0*, *Atcrf11* mutant and *BrCRF11a-*overexpressing transgenic *Arabidopsis p35S:: BrCRF11a*) on MS medium. Relative expressions of *BrCRF11a* and *AtCRF11* in the three types of *Arabidopsis* seedlings were analyzed (Fig. 8b). *BrCRF11a* and *AtCRF11* are orthologous genes, and the similarity of their nucleotide sequences is 81%. It is difficult to design primers for qRT-PCR to distinguish them perfectly. They might affect each other in the expression analysis, but we could find the up-expression and down-expression of the two genes to some extent. After 5 days, an obvious difference was noted in the root lengths of the three types of *Arabidopsis* (Fig. 8a). In the *Atcrf11* mutant, the root length was shorter than that of the wild type, whereas that of *p35S::BrCRF11a* was much longer than that of the wild type. The statistical analysis of the changing root lengths among the three types of *Arabidopsis* showed that the differences were fairly significant (Fig. 8c).

## Discussion

In this study, we identified the *CRFs* (Additional file 2: Table S2) in diploids (*B. rapa*, *B. nigra*, and *B. oleracea*) and amphidiploids (*B. napus* and *B. juncea*) of *Brassica*. Although no new member was found in *B. rapa* [16], we updated some of the species’ information, which is essential to subsequent investigations. In general, most of the new *CRFs*, except for *BnaCRF1a*, *BnaCRF1b*, *BnaCRF2a*, *BnaCRF3a*, and *BjuCRF12a*, do not contain introns (Fig. 2). Among these genes, *BnaCRF1a* and *BjuCRF12a*, are consistent with their original genes *BrCRF1* and *BrCRF12*, respectively, whereas the introns of *BnaCRF1b*, *BnaCRF2a*, and *BnaCRF3a* might emerge after the hybridization of the A and C genomes. In terms of CDS and protein lengths, as well as the molecular weights and pI of the CRFs in the same clade, similar characters were found among the *Brassica* species. In addition, the protein sequences of all the *CRFs* were aligned by ClustalX [48], and similar domains and motifs were identified (Additional file 3: Figure S1) [16, 45]. These results suggest that *CRFs* possess conserved functions among these species, which coincide with the previous result [14].

The *Brassica* diploids underwent an extra whole-genome triplication (WGT) event after diverging from *A. thaliana* [73, 74]. Thus, we calculated the gene retention of every gene and classified them by genome/subgenome and gene name (Additional file 7: Figure S4). This result showed that the gene retentions were similar among genomes but ranged from genes with no ortholog of *AtCRF9* to those with almost no loss of *AtCRF10* ortholog. This biased gene loss following whole-genome duplication is considered to indicate the gene functions [75].

To analyze the selective pressure suffered by the orthologous and paralogous gene pairs, the *Ka*/*Ks* ratios were calculated. *Ka*/*Ks* < 1 reveals a negative selection, whereas *Ka*/*Ks* > 1 indicates a positive selection. Generally speaking, the *Ka*/*Ks* ratios of all the *CRFs* ranged from 0 to 0.5, which suggests a stringent negative selection applied on these genes. Meanwhile, in different *Brassica* species, the *Ka*/*Ks* values of orthologs or paralogs were almost the same (Fig. 4a and c). However, the *Ka*/*Ks* values of the orthologs or paralogs showed an extraordinary difference when classified by gene name. For the orthologous gene pairs between *Brassica* species and *A. thaliana*, the selection pressures on the *CRF7s* were the severest, and the selection pressures on the *CRF11s* were the mildest along the evolution from *A. thaliana* to *Brassica* species (Fig. 4b). For the paralogs within each of the *Brassica* species, the selective pressures on the paralogous genes of *CRF1s* and *CRF8s* were likely stronger, and the paralogs of *CRF6s*, *CRF7s*, *CRF10s*, and *CRF11s* have encountered a milder natural selection within a *Brassica* genome. Meanwhile, the *Ka/Ks* values of orthologous gene pairs were around 0.3, which was higher than those of paralogous gene pairs at about 0.2. This result suggests a weaker natural selection between the *Brassica* species and *A. thaliana* than that in the *Brassica* species alone. Furthermore, meticulous works were carried out to analyze the *Ka*/*Ks* values of the different regions in one gene compared with its ortholog in *A. thaliana* with a sliding window (Additional file 9: Figure S6). We found that most of the *CRFs* in *B. rapa*, *B. oleracea*, and *B. napus* achieved a peak of *Ka*/*Ks* value of more than 1 in the last 100 codons of the C-terminal region. This result implies that the mutations in this region are positively selected, which highly likely embodies their functional differentiation. This opinion is supported by previous research; for instance, the transcriptional activity of AtCRF5 was found to be governed by the C-terminal domain [44], and the C-terminal sequence divergence was correlated with vascular expression [45]. Besides, some *Ka*/*Ks* peaks exceeding 1 were located in the N-termination and in regions close to the AP2 domain; the functions of these regions remain to be elucidated.

Given the wide application of synteny analysis in genome comparison and evolutionary analyses [76, 77], we performed a meticulous synteny analysis for the *CRFs* between the amphidiploids and their parental diploids to ascertain where the *CRFs* in the amphidiploids originate (Additional file 4: Figure S2). As a result, we obtained the original genes in the diploids and their corresponding genes in the amphidiploids (Table 2 and Additional file 5: Table S3). On this basis, the gene rearrangements that occurred after hybridization were discovered, such as *BolCRF3c* with *BnaCRF3d*, *BnaCRF4a* with *BrCRF4a*, *BjuCRF8a* with *BrCRF8a* and so on. Moreover, many genes, such as *BrCRF11b*, *BnaCRF3e*, *BnaCRF4c*, and *BjuCRF5b*, do not possess an original gene or corresponding amphidiploid gene probably because of incomplete genome information or the degeneration of corresponding genes during evolution after hybridization. The *CRF* distribution in the *B. nigra* genome differed from those in *B. rapa* and *B. oleracea*; this result is consistent with a previous conclusion on the close relativity of the *Brassica* A and C genomes and the distinctiveness of the B genome [78, 79]. However, the *CRF* distribution significantly differed between the genome of *B. nigra* and the B subgenome of *B. juncea*. This result implies that the *CRFs* in the B subgenome were considerably influenced by the A subgenome of *B. juncea*. Otherwise, this result was derived from the low sequencing proportion (68%) of the *B. nigra* genome [43]. Notably, four gene pairs (*BnaCRF2b* with *BnaCRF2d*, *BnaCRF6b* with *BnaCRF6c*, *BjuCRF6b* with *BjuCRF6c*, and *BjuCRF12a* with *BjuCRF12b*) were extraordinarily similar to each other and located in different subgenomes of an amphidiploid. On one hand, one gene in the pair may be a copy of the other, which was rearranged [80, 81]. On the other hand, a similar gene may existed in their parental genomes but degenerated after hybridization.

To find clues to the functions of the *CRFs* in *B. nigra*, *B. oleracea*, and *B. napus*, we analyzed the expression patterns of these genes in different organs. All the *CRFs* were constitutively expressed genes, but different relative expression levels were noted in different organs (Fig. 5). The relative expression levels of almost all of the *CRFs* were higer in the flowers and siliques; this observation suggests the genes’ important roles in reproductive development, which was coincident with previous research [20]. Among the four floral organs, many *CRFs* presented higher relative expression levels in the pistils; this finding indicates the link between *CRFs* and pistil development. The crosstalk of cytokinin with other phytohormones is well known to be involved in many plant growth processes [3, 82]. In this present research, numerous *cis*-elements related to hormones were found in the putative promoter region of the *CRFs* (Additional file 10: Table S4 and Additional file 11: Table S5). Additionally, most *CRFs* were found to respond to cytokinin (6-BA), auxin (NAA), and ABA treatments, although the response patterns are dissimilar (Fig. 6). Such result agrees with those of the *CRFs* that highly participate in the crosstalk between cytokinin and other hormones, such as auxin, ABA, and salicylic acid [20, 25, 83]. To date, numbers of AP2/ERF TFs have been identified to be related to abiotic stress responses in various plant species [70, 71]. Since a subfamily of the AP2/ERF family consist of ERF TFs, some members of the ERF subfamily have been proven to respond to many abiotic stresses, such as high salinity and drought [84]. Thus, CRFs, as Group VI and VI-L members of the ERF subfamily, also share the capability to respond to abiotic stresses [16, 23]. A similar result was obtained in this study (Fig. 7), and *cis*-elements related to stresses in the putative promoter region of the *CRFs* (Additional file 10: Table S4 and Additional file 11: Table S5) also supported our results.

*B. napus* and *B. juncea* are known to be formed ~ 7500 years or 0.038–0.055 MYA by the hybridization between *B. rapa* and *B. oleracea*/*B. nigra* [40, 43]; such event is far behind the divergence time of *B. rapa* (13–17 MYA) [42]. Although the evolution time is relatively shorter for the amphidiploid *B. napus*, the *CRFs* in this genome considerably differ from those of its parental genomes. For example, only 3 of 16 CRF gene pairs between *B. napus* and *B. oleracea* exhibited metastable expression patterns in at least two situations in this research. The orthologous gene pairs among the three diploids also shared low similarities in expression profiles [16]. All these results indicate that *CRFs* likely serve species-specific functions despite their numerous common functions.

In our previous study [16], we found that the relative expression level of *BrCRF11a* in the root of *B. rapa* was higher. In this present study, we found that the relative expression levels of *BolCRF11*, *BniCRF11a*, and *BniCRF11b* in the root were also higher (Fig. 5). These results indicate that the *CRF11s* may be related to the root growth. We analyzed the functions of *AtCRF11* and *BrCRF11a* in the *Arabidopsis* root. Knocking out *AtCRF11* inhibited primary root growth, whereas overexpressing *BrCRF11a* in *Arabidopsis* resulted in primary root growth promotion. These findings confirmed our hypothesis and agreed with those of previous works, which indicated the expression or function of the *CRF* genes in the root in many species, such as *A. thaliana* [19], *S. lycopersicum* [24], and *B. rapa* [16]. However, the relative expression levels of *BnaCRF11a* and *BnaCRF11b* in the root were lower. It needs further study to analyze the functions of *CRF11s* in *B. rapa*, *B. oleracea*, and *B. napus*, to find whether the fuctional differentiation exists and to explore their precise biological roles.

## Conclusion

In this study, we characterized 141 *CRF* genes in three diploids and two amphidiploids of *Brassica* U-triangle species. On the basis of the comparisons among their sequences and expression patterns, we analyzed the functional inheritance and differentiation of *CRFs* among the species during the evolution. Our results showed the close relativity of the *Brassica* A and C genomes and the distinctiveness of the B genome, and the B subgenome was considerably influenced by the A subgenome of *B. juncea*.. Furthermore, we firstly discovered the function of a Clade V *CRF*, *CRF11*, related to root growth. This study provided insights into the functional genomics and evolutionary biology of plants and obtained useful information for fine farming and improved breeding.

## Additional files

Additional file 1: **Table S1.** Primers used for qRT-PCR. (XLS 47 kb)

Additional file 2: **Table S2.** Characterization of *CRFs* in *Brassica* species. (DOC 255 kb)

Additional file 3: **Figure S1.** Conserve motif alignment of three types CRFs. The figure was obtained by ClustalX and WedLogo 3 (http://weblogo.threeplusone.com/). (DOC 3169 kb)

Additional file 4: **Figure S2.** Synteny analysis of *CRFs* among *Brassica* species. (A) Alignment between genes in diploid and in allotetraploid. (B) Alignment between similar genes in allotetraploid. Gene models are colored in grey (gene) and green (CDS). Red blocks indicate the high-scoring segment pair (HSP), and pink links show the connectors between HSP.The figure was formed on GEvo (https://genomevolution.org/CoGe/GEvo.pl). (TIF 8633 kb)

Additional file 5: **Table S3.** List of origin genes in *B. rapa, B. nigra* and amphidiploid genes in *B. napus, B. juncea*. High-similarity genes are marked in bold. (DOC 58 kb)

Additional file 6: **Figure S3.** Chromosomal mapping of *CRFs* in *B. nigra* and *B. juncea*. The *CRFs* in *B. nigra* (A) and *B. juncea* (B) are shown except those located on the scaffolds. The locations were shown on the left of the chromosomes, whereas the gene names were on the right. The arrows next to gene names show the direction of transcription. The bar indicates the size of 5 Mb. (TIF 9375 kb)

Additional file 7: **Figure S4.** Box-plots of gene retention ratio. Statistics by genomes (A) and by gene names (B) are shown. The Violin plots indicate the number of the same value. (TIF 3464 kb)

Additional file 8: **Figure S5.** Gene retention ratios of *CRFs* in the three sub-genomes of *B. rapa*, *B. oleracea*, and *B. napus*. (TIF 1003 kb)

Additional file 9: **Figure S6.** *Ka/Ks* values of CRF orthologous gene pairs of *Br-At, Bol-At* and *Bna-At* over a sliding window of 20 codons. The x-axis indicates the starting codon of sliding window. The y-axis shows the *Ka/Ks* values. (DOC 1218 kb)

Additional file 10: **Table S4.** Elements relate to hormone and stress in the promotor of *CRFs* predicted by PLACE.Table S5. Elements relate to hormone and stress in the promotor of *CRFs* predicted by PlantCARE. (XLS 39 kb)

Additional file 11: **Table S5.** Elements relate to hormone and stress in the promotor of *CRFs* predicted by PlantCARE. (XLS 37 kb)

## Abbreviations

6-BA: 6-Benzylaminopurine
*A.*: *Arabidopsis*
ABA: Abscisic acid
ARRs: *Arabidopsis* response regulators
*B.*: *Brassica*
*CRF*: *Cytokinin Response Factor*
HPs: His-containing phosphotransfer proteins
*Ka*: Non-synonymous substitution rates
*Ks*: Synonymous substitution rates
MS: Murashige and Skoog
MYA: Million years ago
NAA: 1-naphthylacetic acid
qRT-PCR: Quantitative reverse-transcription PCR
*S.*: *Solanum*
TFs: Transcriptional factors
WGT: Whole-genome triplication
