# Supplementary material for: Comparative analysis of cytokinin response factors in Brassica diploids and amphidiploids and insights into the evolution of Brassica species
Source: BMC Genomics. 2018 Oct 3;19:728. doi: 10.1186/s12864-018-5114-y (PMC6171139; doi:10.1186/s12864-018-5114-y)
Supplement: Supplementary file 2 — Table S2. Characterization of CRFs in Brassica species. (DOC 255 kb) [file 12864_2018_5114_MOESM2_ESM.doc]

**Table S2 Characterization of *CRFs* in *Brassica* species.**

**Characterization of *CRFs* in *B. rapa***.

| **Gene^a^** | **Locus^b^** | **Chr^c^** | **Position^d^ (5′-3′ bp)** | **ORF length^e^ (bp)** | **Deduced polypeptide^f^** | | |
| --- | --- | --- | --- | --- | --- | --- | --- |
|  |  |  |  |  | **Length (aa)** | **MW (kDa)** | **PI** |
| *BrCRF1* | Bra000736 | A03 | 12771319-12772220(-) | 822 | 273 | 29.9 | 5.12 |
| *BrCRF2a* | Bra019270 | A03 | 25376708-25377640(+) | 933 | 310 | 34.5 | 5.38 |
| *BrCRF2b* | Bra013730 | A01 | 7482052-7482999(+) | 948 | 315 | 34.9 | 5.34 |
| *BrCRF3a* | Bra003068 | A10 | 5545504-5546598(-) | 1095 | 364 | 41.4 | 4.71 |
| *BrCRF3b* | Bra029079 | A03 | 6251090-6252121(+) | 1032 | 343 | 39.0 | 4.60 |
| *BrCRF3c* | Bra022643 | A02 | 7303322-7304380(+) | 1059 | 352 | 39.7 | 4.56 |
| *BrCRF4a* | Bra040839 | A08 | 11273407-11274384(-) | 978 | 325 | 36.9 | 4.84 |
| *BrCRF4b* | Bra010389 | A08 | 13461939-13462925(+) | 987 | 328 | 37.4 | 4.81 |
| *BrCRF4c* | Bra026295 | A01 | 9962055-9963068(-) | 1014 | 337 | 38.2 | 4.96 |
| *BrCR5* | Bra004548 | A05 | 758917-759726(-) | 810 | 269 | 30.0 | 7.62 |
| *BrCRF6* | Bra003462 | A07 | 16595971-16596822(+） | 852 | 283 | 31.8 | 5.43 |
| *BrCRF7a* | Bra012352 | A07 | 10518683-10519126(+) | 444 | 147 | 16.1 | 9.93 |
| *BrCRF7b* | Bra016367 | A08 | 17301404-17301880(+) | 477 | 158 | 17.3 | 9.79 |
| *BrCRF8a* | Bra003936 | A07 | 19114985-19115482(-) | 498 | 165 | 18.5 | 9.69 |
| *BrCRF8b* | Bra007963 | A02 | 11153118-11153621(+) | 504 | 167 | 18.6 | 9.58 |
| *BrCRF10a* | Bra033923 | A02 | 9919687-9920637(-) | 951 | 316 | 34.7 | 4.62 |
| *BrCRF10b* | Bra004318 | A07 | 21224041-21224955(-) | 915 | 304 | 33.8 | 4.91 |
| *BrCRF10c* | Bra004033 | A07 | 19701034-19701987(+) | 954 | 317 | 35.2 | 4.82 |
| *BrCRF11a* | Bra025170 | A06 | 21898085-21899041(-) | 957 | 318 | 35.2 | 5.03 |
| *BrCRF11b* | Bra036360 | A02 | 21201771-21202616(+) | 846 | 281 | 31.6 | 5.17 |
| *BrCRF12* | Bra024743 | A09 | 24340128- 24341221(-) | 1005 | 334 | 37.4 | 5.17 |

^a^Names given to *BrCRFs* in this work.

^b^Locus represented by the *B. rapa* genome database.

^c^Chromosomal localization of the *BrCRFs*.

^d^Position of *BrCRFs* on the chromosome, +/- represents the direction of transcription.

^e^Length of open reading frame in base pairs.

^f^Length (number of amino acids), molecular weight (kDa), and isoelectric point (pI) of the deduced polypeptide.

Characterization of *CRFs* in *B. nigra*.

| **Gene^a^** | **Locus^b^** | **Chr^c^** | **Position^d^ (5′-3′ bp)** | **ORF length^e^ (bp)** | **Deduced polypeptide^f^** | | |
| --- | --- | --- | --- | --- | --- | --- | --- |
|  |  |  |  |  | **Length (aa)** | **MW (kDa)** | **PI** |
| *BniCRF1* | BniB027724 | B03 | 23108560-23109450(+) | 891 | 296 | 32.5 | 4.76 |
| *BniCRF2a* | BniB002161 | -- | 2736854-2737810(-) | 957 | 318 | 35.3 | 5.56 |
| *BniCRF2b* | BniB013242 | B02 | 37839212-37840204(+) | 993 | 330 | 36.2 | 5.03 |
| *BniCRF3a* | BniB006006 | -- | 476218-477303(-) | 1086 | 361 | 41.0 | 4.83 |
| *BniCRF3b* | BniB046682 | B03 | 36178083-36179129(-) | 1047 | 348 | 39.3 | 4.61 |
| *BniCRF3c* | BniB006608 | B02 | 26737499-26738575(+) | 1077 | 358 | 40.3 | 4.81 |
| *BniCRF4a* | BniB002637 | -- | 1755467-1756468(+) | 1002 | 333 | 38.1 | 5.07 |
| *BniCRF4b* | BniB015776 | B07 | 37181529-37182485(-) | 957 | 318 | 36.2 | 4.83 |
| *BniCRF4c* | BniB018756 | B02 | 39812283-39813278(-) | 996 | 331 | 37.4 | 4.95 |
| *BniCR5a* | BniB044117 | B01 | 28426982-28427899(-) | 918 | 305 | 34.0 | 5.78 |
| *BniCR5b* | BniB048901 | B03 | 26380251-26381105(-) | 855 | 284 | 31.8 | 4.90 |
| *BniCRF6a* | BniB019738 | B04 | 24450432-24451313(-) | 882 | 293 | 33.2 | 5.21 |
| *BniCRF6b* | BniB029687 | B03 | 45218-46066(+) | 849 | 282 | 31.9 | 4.86 |
| *BniCRF6c* | BniB029674 | B03 | 97124-97954(-) | 831 | 276 | 31.1 | 5.28 |
| *BmiCRF7a* | BniB039746 | B07 | 26702328-26702789(-) | 462 | 153 | 16.8 | 9.47 |
| *BniCRF7b* | BniB048344 | B07 | 34235408-34235869(-) | 462 | 153 | 17.1 | 9.73 |
| *BniCRF8a* | BniB042646 | B07 | 25652522-25653013(-) | 492 | 163 | 18.2 | 9.65 |
| *BniCRF8b* | BniB046509 | -- | 739178-739675(+) | 498 | 165 | 18.3 | 9.80 |
| *BniCRF10a* | BniB033638 | -- | 926720-927646(+) | 927 | 308 | 34.0 | 4.70 |
| *BniCRF10b* | BniB032011 | B07 | 17520033-17520998(+) | 966 | 321 | 35.8 | 4.95 |
| *BniCRF10c* | BniB035458 | B07 | 19946943-19947899(+) | 957 | 318 | 35.5 | 4.81 |
| *BniCRF10d* | BniB033637 | -- | 915273-916199(+) | 927 | 308 | 34.0 | 4.70 |
| *BniCRF11a* | BniB025326 | B08 | 36463715-36464701(+) | 987 | 328 | 35.9 | 4.96 |
| *BniCRF11b* | BniB027210 | B06 | 29328971-29329750(+) | 780 | 259 | 29.3 | 5.26 |

^a^Names given to *BniCRFs* in this work.

^b^Locus represented by the *B. nigra* genome database.

^c^Chromosomal localization of the *BniCRFs*.

^d^Position of *BniCRFs* on the chromosome or contig, +/- represents the direction of transcription.

^e^Length of open reading frame in base pairs.

^f^Length (number of amino acids), molecular weight (kDa), and isoelectric point (pI) of the deduced polypeptide.

“--”Gene locates on contig or scaffold, and the number is not shown.

Characterization of *CRFs* in *B. oleracea*.

| **Gene^a^** | **Locus^b^** | **Chr^c^** | **Position^d^ (5′-3′ bp)** | **ORF length^e^ (bp)** | **Deduced polypeptide^f^** | | |
| --- | --- | --- | --- | --- | --- | --- | --- |
|  |  |  |  |  | **Length (aa)** | **MW (kDa)** | **PI** |
| *BolCRF1* | Bol025704 | C03 | 16475821-16476717(-) | 897 | 298 | 32.6 | 4.87 |
| *BolCRF2* | Bol042124 | C07 | 43222902-43223846(+) | 945 | 314 | 34.7 | 5.67 |
| *BolCRF3a* | Bol028000 | C03 | 7146494-7147531(+) | 1038 | 345 | 39.3 | 4.57 |
| *BolCRF3b* | Bol030298 | C09 | 20174758-20175846(-) | 1089 | 362 | 41.2 | 4.70 |
| *BolCRF3c* | Bol017173 | C07 | 25504535-25505584(-) | 1050 | 349 | 39.3 | 4.63 |
| *BolCRF4a* | Bol042364 | C07 | 44576019-44576993(-) | 975 | 324 | 36.9 | 4.75 |
| *BolCRF4b* | Bol021157 | C08 | 15709929-15710915(-) | 987 | 328 | 37.3 | 4.82 |
| *BolCRF4c* | Bol013128 | C01 | 14146413-14147426(-) | 1014 | 337 | 38.3 | 4.96 |
| *BolCRF5* | Bol000916 | -- | 14202-15023(+) | 822 | 273 | 30.5 | 7.62 |
| *BolCRF6a* | Bol010878 | C06 | 16120210-16121073(+) | 864 | 287 | 32.0 | 5.22 |
| *BolCRF6b* | Bol045665 | C08 | 32792030-32792893(+) | 864 | 287 | 32.5 | 4.72 |
| *BolCRF7a* | Bol012318 | C07 | 23754753-23755220(+) | 468 | 155 | 17.2 | 10.00 |
| *BolCRF7b* | Bol012606 | -- | 32755-33240(+) | 486 | 161 | 17.7 | 10.00 |
| *BolCRF8a* | Bol002344 | -- | 34017-34514(+) | 498 | 165 | 18.4 | 9.84 |
| *BolCRF8b* | Bol034960 | C02 | 14498309-14498800(+) | 492 | 163 | 18.2 | 9.30 |
| *BolCRF10a* | Bol003308 | -- | 86301-87254(-) | 954 | 317 | 35.3 | 4.77 |
| *BolCRF10b* | Bol024017 | C06 | 8206823-8207728(+) | 906 | 301 | 33.6 | 4.97 |
| *BolCRF11* | Bol042768 | C07 | 31913467-31914408(+) | 942 | 313 | 34.6 | 5.01 |

^a^Names given to *BolCRFs* in this work.

^b^Locus represented by the *B. oleracea* genome database.

^c^Chromosomal localization of the *BolCRFs*.

^d^Position of *BolCRFs* on the chromosome or contig, +/- represents the direction of transcription.

^e^Length of open reading frame in base pairs.

^f^Length (number of amino acids), molecular weight (kDa), and isoelectric point (pI) of the deduced polypeptide.

“--”Gene locates on contig or scaffold, and the number is not shown.

Characterization of *CRFs* in *B. napus*.

| **Gene^a^** | **Locus^b^** | **Chr^c^** | **Position^d^ (5′-3′ bp)** | **ORF length^e^ (bp)** | **Deduced polypeptide^f^** | | |
| --- | --- | --- | --- | --- | --- | --- | --- |
|  |  |  |  |  | **Length (aa)** | **MW (kDa)** | **PI** |
| *BnaCRF1a* | BnaA03g24540D | A03 | 11821472-11822377 (+) | 834 | 277 | 30.3 | 5.12 |
| *BnaCRF1b* | BnaC03g29310D | C03 | 17510128-17511033 (-) | 834 | 277 | 30.2 | 5.09 |
| *BnaCRF2a* | BnaA03g46240D | A03 | 23700255-23701187 (+) | 891 | 296 | 33.0 | 5.50 |
| *BnaCRF2b* | BnaA01g13420D | A01 | 6830329-6831276 (+) | 948 | 315 | 34.9 | 5.34 |
| *BnaCRF2c* | BnaC07g38490D | C07 | 39823344-39824288(+) | 945 | 314 | 34.7 | 5.67 |
| *BnaCRF2d* | BnaC01g15610D | C01 | 10709498-10710466 (+) | 969 | 322 | 35.5 | 5.10 |
| *BnaCRF3a* | BnaC03g14960D | C03 | 7314912-7316028 (+) | 1014 | 337 | 38.3 | 4.64 |
| *BnaCRF3b* | BnaA03g12320D | A03 | 5607387-5608495 (+) | 1029 | 342 | 38.9 | 4.67 |
| *BnaCRF3c* | BnaA02g10340D | A02 | 5285382-5286513 (+) | 1056 | 351 | 39.7 | 4.62 |
| *BnaCRF3d* | BnaC02g14430D | C02 | 9853122-9854171 (+) | 1050 | 349 | 39.4 | 4.63 |
| *BnaCRF3e* | BnaCnng38100D | Cnn | 36638520-36639608 (-) | 1089 | 362 | 41.2 | 4.71 |
| *BnaCRF4a* | BnaA03g48910D | A03 | 25137422-25138399 (-) | 978 | 325 | 36.9 | 4.84 |
| *BnaCRF4b* | BnaA08g13940D | A08 | 11929169-11930155 (+) | 987 | 328 | 37.3 | 4.81 |
| *BnaCRF4c* | BnaAnng36370D | Ann | 41280315-41281328(+) | 1014 | 337 | 38.3 | 4.95 |
| *BnaCRF4d* | BnaC08g12710D | C08 | 17838966-17839952(-) | 987 | 328 | 37.2 | 4.78 |
| *BnaCRF4e* | BnaCnng73120D | Cnn | 73592892-73593905(+) | 1014 | 337 | 38.3 | 4.96 |
| *BnaCRF4f* | BnaC07g41100D | C07 | 41105587-41106561 (-) | 975 | 324 | 37.0 | 4.75 |
| *BnaCRF5a* | BnaC04g01080D | C04 | 904940-905783(+) | 822 | 273 | 30.5 | 7.62 |
| *BnaCRF5b* | BnaA05g01500D | A05 | 869266-870245(-) | 810 | 269 | 30.0 | 7.62 |
| *BnaCRF6a* | BnaA07g38140D | A07 | 1494132-1494986(-) | 855 | 284 | 31.7 | 5.73 |
| *BnaCRF6b* | BnaC08g31500D | C08 | 30864603-30865635(+) | 867 | 288 | 32.5 | 4.65 |
| *BnaCRF6c* | BnaA09g39350D | A09 | 27899462-27900313(+) | 852 | 283 | 32.1 | 4.77 |
| *BnaCRF6d* | BnaC06g42850D | C06 | 2398812-2399675(-) | 864 | 287 | 32.0 | 5.30 |
| *BnaCRF7a* | BnaC07g13400D | C07 | 19005195-19005662(+) | 468 | 155 | 17.1 | 9.90 |
| *BnaCRF7b* | BnaC08g06130D | C08 | 8645760-8646424(-) | 486 | 161 | 17.7 | 10.0 |
| *BnaCRF7c* | BnaAnng35170D | Ann | 39965631-39966136(+) | 444 | 147 | 16.3 | 9.93 |
| *BnaCRF7d* | BnaA08g20700D | A08 | 15517996-15519030(+) | 477 | 158 | 17.4 | 9.91 |
| *BnaCRF8a* | BnaA07g23650D | A07 | 17743299-17743947(-) | 498 | 165 | 18.4 | 9.80 |
| *BnaCRF8b* | BnaC02g20580D | C02 | 17111886-17112714(+) | 495 | 164 | 18.2 | 9.44 |
| *BnaCRF8c* | BnaCnng17120D | Cnn | 16069826-16070323(-) | 498 | 165 | 18.4 | 9.84 |

Continued previous page

| *BnaCRF8d* | BnaA02g35550D | A02 | 356250-357083(+) | 504 | 167 | 18.6 | 9.58 |
| --- | --- | --- | --- | --- | --- | --- | --- |
| *BnaCRF10a* | BnaA02g13950D | A02 | 7767126-7768335(-) | 951 | 316 | 34.7 | 4.62 |
| *BnaCRF10b* | BnaA07g27210D | A07 | 19792908-19794008(-) | 915 | 304 | 33.7 | 4.91 |
| *BnaCRF10c* | BnaA07g24590D | A07 | 18399051-18400169(+) | 948 | 315 | 35.1 | 4.80 |
| *BnaCRF10d* | BnaAnng20260D | Ann | 22052997-22054120(+) | 942 | 313 | 34.7 | 4.68 |
| *BnaCRF10e* | BnaC06g30140D | C06 | 30976375-30977480(-) | 906 | 301 | 33.6 | 4.97 |
| *BnaCRF10f* | BnaC06g25810D | C06 | 27493975-27494928(+) | 954 | 317 | 35.2 | 4.77 |
| *BnaCRF11a* | BnaA06g33270D | A06 | 22013904-22015179(-) | 954 | 317 | 35.1 | 5.03 |
| *BnaCRF11b* | BnaC07g23080D | C07 | 29726650-29727918(+) | 942 | 313 | 34.6 | 5.01 |
| *BnaCRF12* | BnaC05g20650D | C05 | 14045421-14046333(+) | 747 | 248 | 27.6 | 5.52 |

^a^Names given to *BnaCRFs* in this work.

^b^Locus represented by the *B. napus* genome database.

^c^Chromosomal localization of the *BnaCRFs*.

^d^Position of *BnaCRFs* on the chromosome, +/- represents the direction of transcription.

^e^Length of open reading frame in base pairs.

^f^Length (number of amino acids), molecular weight (kDa), and isoelectric point (pI) of the deduced polypeptide.

Characterization of *CRFs* in *B. juncea*.

| **Gene^a^** | **Locus^b^** | **Chr^c^** | **Position^d^ (5′-3′ bp)** | **ORF length^e^ (bp)** | **Deduced polypeptide^f^** | | |
| --- | --- | --- | --- | --- | --- | --- | --- |
|  |  |  |  |  | **Length (aa)** | **MW (kDa)** | **PI** |
| *BjuCRF1a* | BjuA011261 | A03 | 15688834-15689724(-) | 891 | 297 | 32.6 | 4.89 |
| *BjuCRF1b* | BjuB041440 | B08 | 33981372-33982259(-) | 888 | 296 | 32.5 | 4.80 |
| *BjuCRF2a* | BjuA012373 | A03 | 32810206-32811135(+) | 930 | 310 | 34.5 | 5.51 |
| *BjuCRF2b* | BjuB014183 | B05 | 8616318-8617304(-) | 987 | 329 | 36.1 | 5.02 |
| *BjuCRF2c* | BjuO006975 | -- | -- | 957 | 318 | 35.4 | 5.89 |
| *BjuCRF2d* | BjuA004117 | A01 | 8721943-8722887(+) | 945 | 315 | 34.9 | 5.34 |
| *BjuCRF3a* | BjuB047677 | B02 | 23130092-23131174(-) | 1083 | 361 | 40.9 | 4.86 |
| *BjuCRF3b* | BjuA009734 | A03 | 7833456-7834484(+) | 1029 | 342 | 38.9 | 4.67 |
| *BjuCRF3c* | BjuA006949 | A02 | 8486497- 8487552(+) | 1056 | 352 | 39.7 | 4.61 |
| *BjuCRF3d* | BjuA038161 | A10 | 8101428-8102504(-) | 1077 | 359 | 40.7 | 4.74 |
| *BjuCRF3e* | BjuB041132 | B08 | 15468900-15469949(+) | 1050 | 350 | 39.5 | 4.59 |
| *BjuCRF3f* | BjuO004155 | -- | 97358-98431(-) | 1074 | 358 | 40.4 | 4.81 |
| *BjuCRF4* | BjuO005474 | -- | 1358-2359 (+) | 1002 | 333 | 37.9 | 5.26 |
| *BjuCRF5a* | BjuO002131 | -- | 140329-141207(-) | 879 | 293 | 32.8 | 5.67 |
| *BjuCRF5b* | BjuA042143 | A03 | 24244376-24245245(-) | 870 | 290 | 32.2 | 5.01 |
| *BjuCRF5c* | BjuA043006 | A05 | 776022-776828(-) | 807 | 269 | 30.0 | 7.62 |
| *BjuCRF6a* | BjuA026784 | A07 | 24714523-24715374(+) | 852 | 284 | 31.9 | 5.44 |
| *BjuCRF6b* | BjuB005038 | B08 | 69825252-69826088(-) | 837 | 279 | 31.5 | 4.80 |
| *BjuCRF6c* | BjuA036267 | A09 | 47096790-47097638(+) | 849 | 283 | 32.1 | 4.77 |
| *BjuCRF6d* | BjuB022238 | B06 | 23772463-23773341(-) | 879 | 293 | 33.1 | 5.01 |
| *BjuCRF7a* | BjuA026003 | A07 | 15942515-15942955(+) | 441 | 147 | 16.3 | 9.99 |
| *BjuCRF7b* | BjuA046696 | A08 | 22125925-22126398(-) | 474 | 158 | 17.5 | 9.79 |
| *BjuCRF7c* | BjuB002985 | B03 | 7401514-7401984(+) | 471 | 156 | 17.4 | 9.95 |
| *BjuCRF8a* | BjuB047433 | B03 | 25619838-25620332(-) | 495 | 165 | 18.5 | 9.69 |
| *BjuCRF8b* | BjuA007403 | A02 | 8486497-8487552(+) | 501 | 167 | 18.6 | 9.58 |
| *BjuCRF8c* | BjuB005433 | B03 | 25804692-25805180(+) | 489 | 163 | 18.2 | 9.65 |
| *BjuCRF8d* | BjuB047642 | B02 | 15364288-15364785(+) | 498 | 166 | 18.3 | 9.45 |
| *BjuCRF10a* | BjuA045416 | A02 | 11398566 -11399510(-) | 945 | 315 | 34.7 | 4.58 |
| *BjuCRF10b* | BjuA046590 | A07 | 29616801-29617715(-) | 915 | 305 | 33.8 | 4.91 |
| *BjuCRF10c* | BjuA027207 | A07 | 27875328-27876272(+) | 945 | 315 | 35.1 | 4.81 |

Continued previous page

| *BjuCRF10d* | BjuB010804 | B05 | 54652308-54653231(-) | 924 | 308 | 34.1 | 4.70 |
| --- | --- | --- | --- | --- | --- | --- | --- |
| *BjuCRF10e* | BjuO012146 | -- | 1159559-1160521(+) | 963 | 321 | 35.8 | 4.95 |
| *BjuCRF10f* | BjuB000729 | B03 | 28521880-28522845(-) | 966 | 322 | 35.7 | 4.81 |
| *BjuCRF11a* | BjuA024408 | A06 | 26025846-26026799(-) | 954 | 318 | 35.2 | 5.03 |
| *BjuCRF11b* | BjuA030646 | A02 | 31240757-31241599(+) | 843 | 281 | 31.6 | 5.17 |
| *BjuCRF11c* | BjuB027369 | B04 | 2982342-2983118(-) | 777 | 259 | 29.2 | 5.38 |
| *BjuCRF12a* | BjuA010916 | A09 | 34830076-34831169(-) | 1005 | 334 | 37.4 | 5.17 |
| *BjuCRF12b* | BjuB045065 | B07 | 27858416-27859566(+) | 1151 | 318 | 34.9 | 5.50 |

^a^Names given to *BjuCRFs* in this work.

^b^Locus represented by the *B. juncea* genome database.

^c^Chromosomal localization of the *BjuCRFs*.

^d^Position of *BjuCRFs* on the chromosome or contig, +/- represents the direction of transcription.

^e^Length of open reading frame in base pairs.

^f^Length (number of amino acids), molecular weight (kDa), and isoelectric point (pI) of the deduced polypeptide.

“--”Gene locates on contig or scaffold, and the number is not shown.
