# Supplementary material for: Comparative analysis of cytokinin response factors in Brassica diploids and amphidiploids and insights into the evolution of Brassica species
Source: BMC Genomics. 2018 Oct 3;19:728. doi: 10.1186/s12864-018-5114-y (PMC6171139; doi:10.1186/s12864-018-5114-y)

**Figure S1. Conserve motif alignment of three types CRFs**

**Type A:** include CladeⅠⅡⅢ( CRF1~6s)


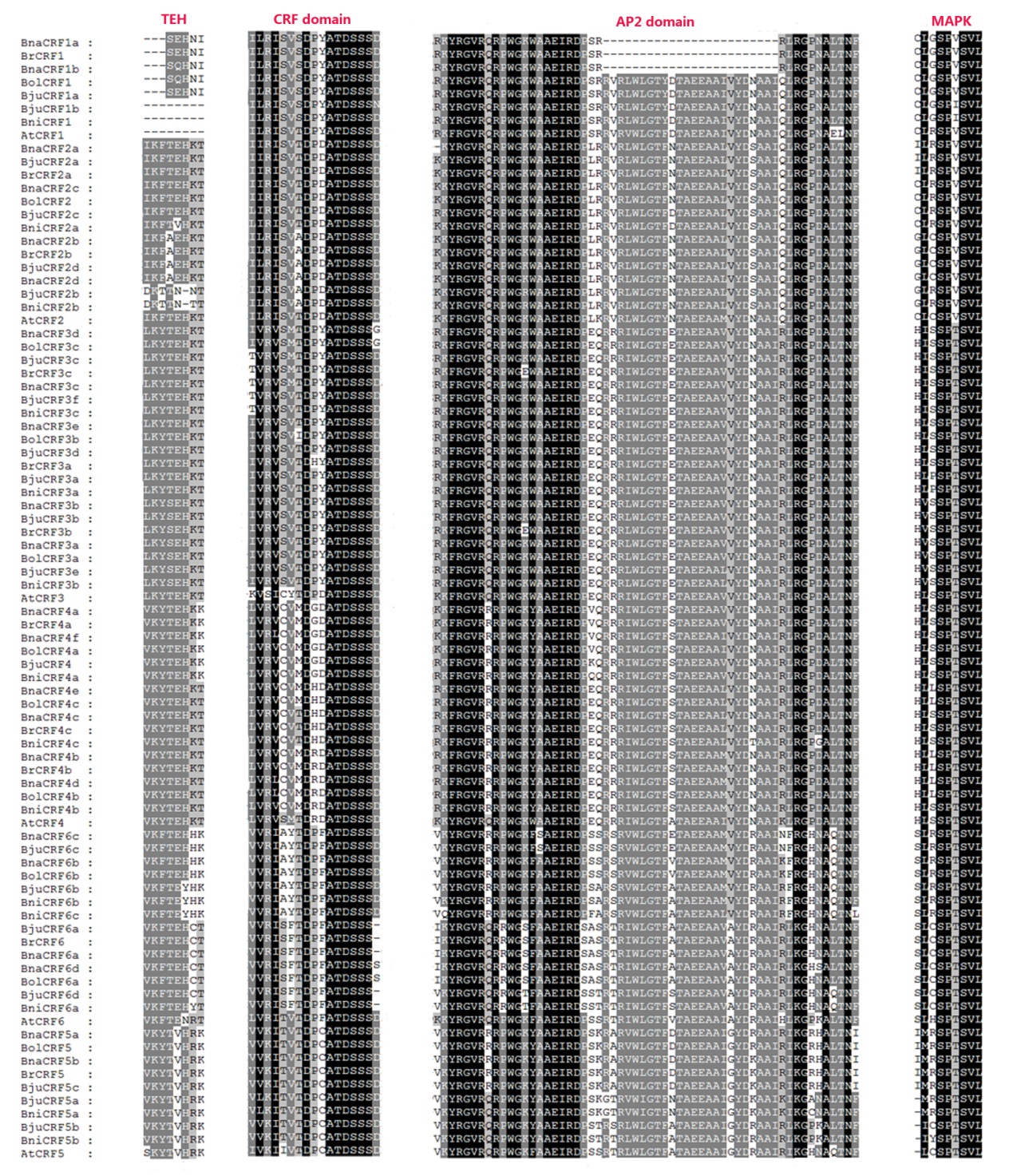


**Type B:** include CladeⅣ( CRF7s,CRF8s)


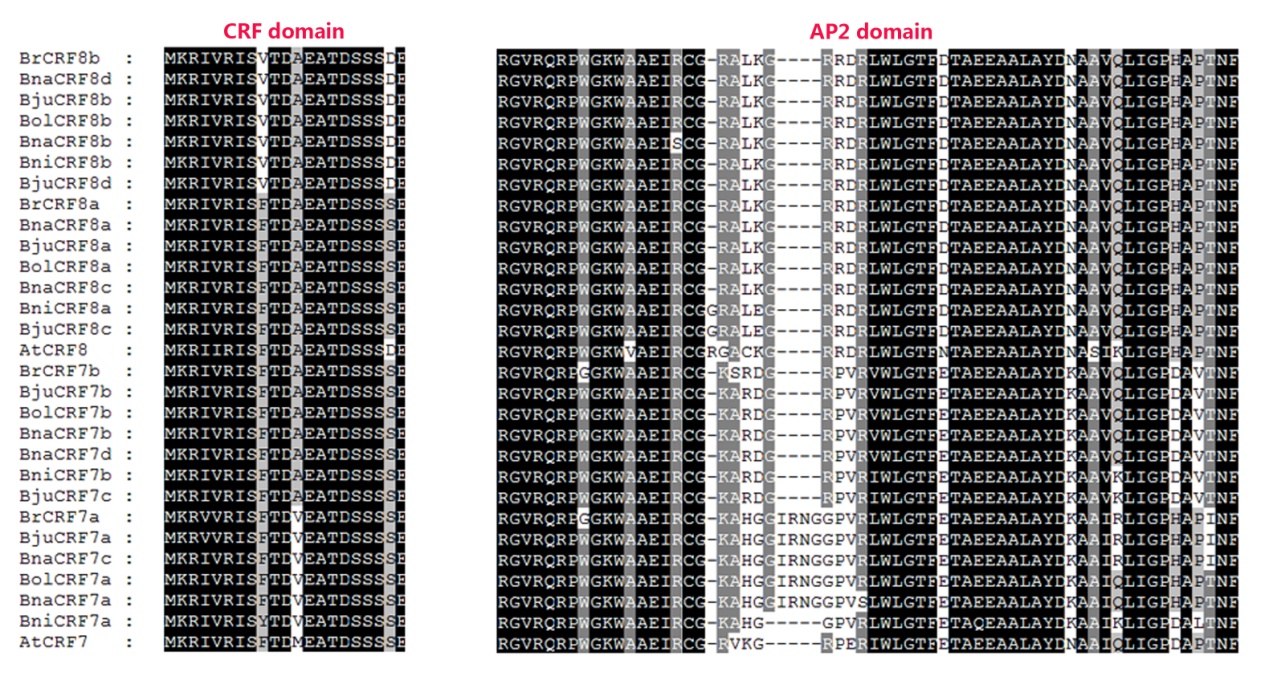


**Type C:** include CladeⅤ( CRF9~12)


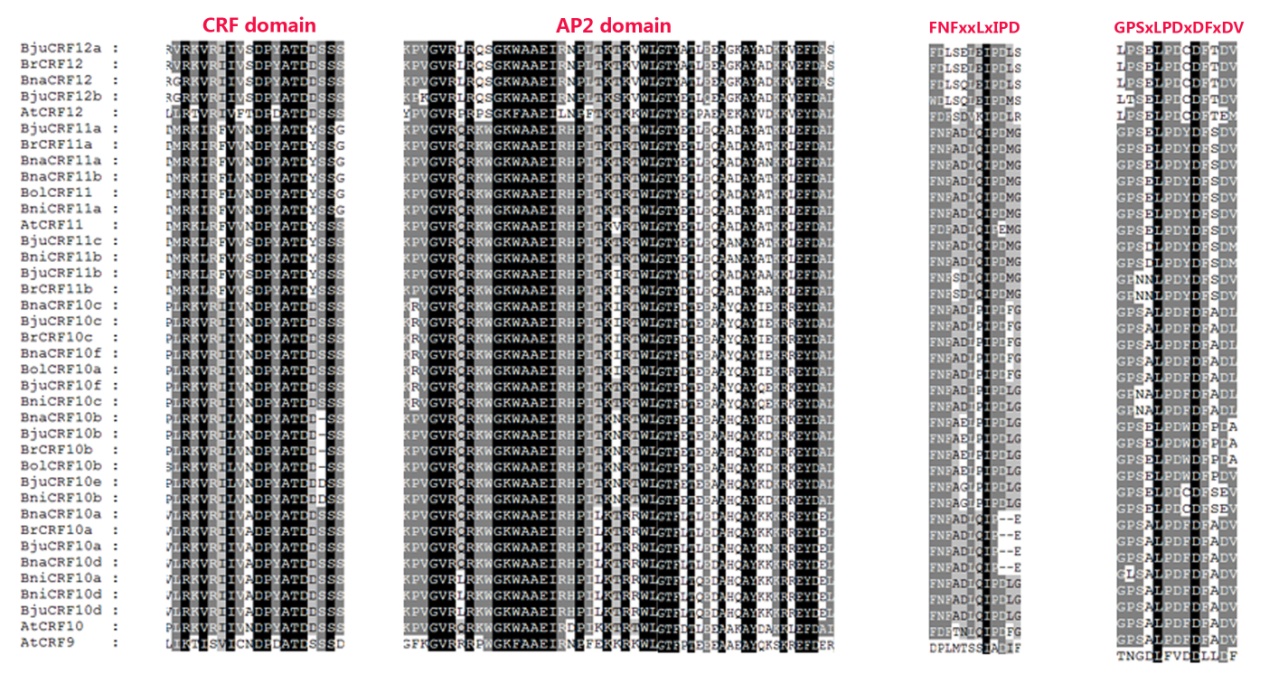


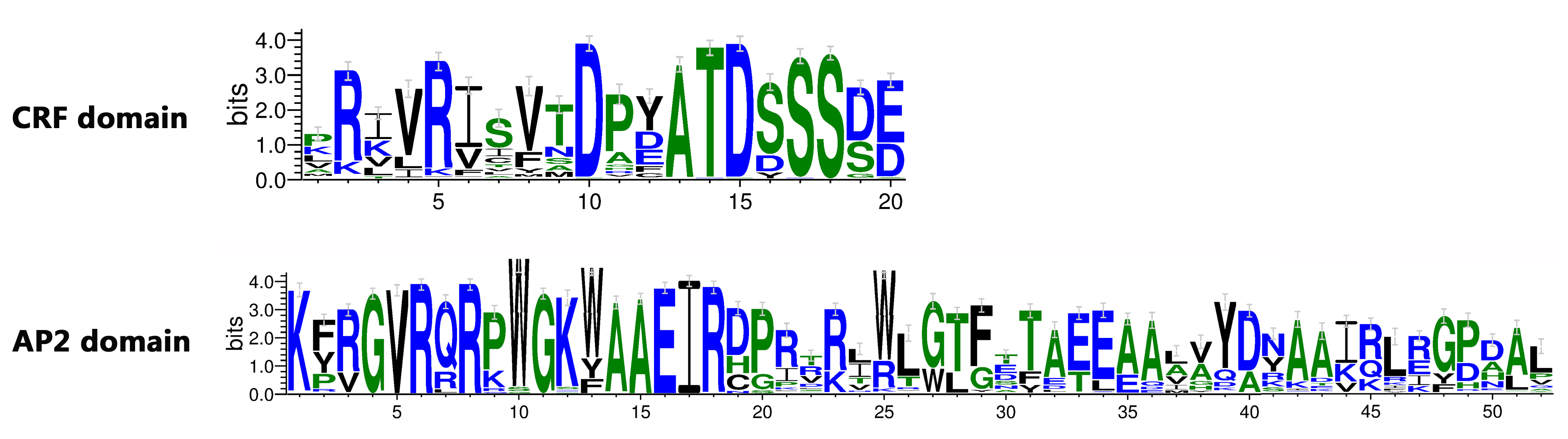

Supplement: Supplementary file 3 — Figure S1. Conserve motif alignment of three types CRFs. The figure was obtained by ClustalX and WedLogo 3 (http://weblogo.threeplusone.com/). (DOC 3169 kb) [file 12864_2018_5114_MOESM3_ESM.doc]
