# Supplementary figures and images for: Comparative analysis of cytokinin response factors in Brassica diploids and amphidiploids and insights into the evolution of Brassica species
Source: BMC Genomics. 2018 Oct 3;19:728. doi: 10.1186/s12864-018-5114-y (PMC6171139; doi:10.1186/s12864-018-5114-y)

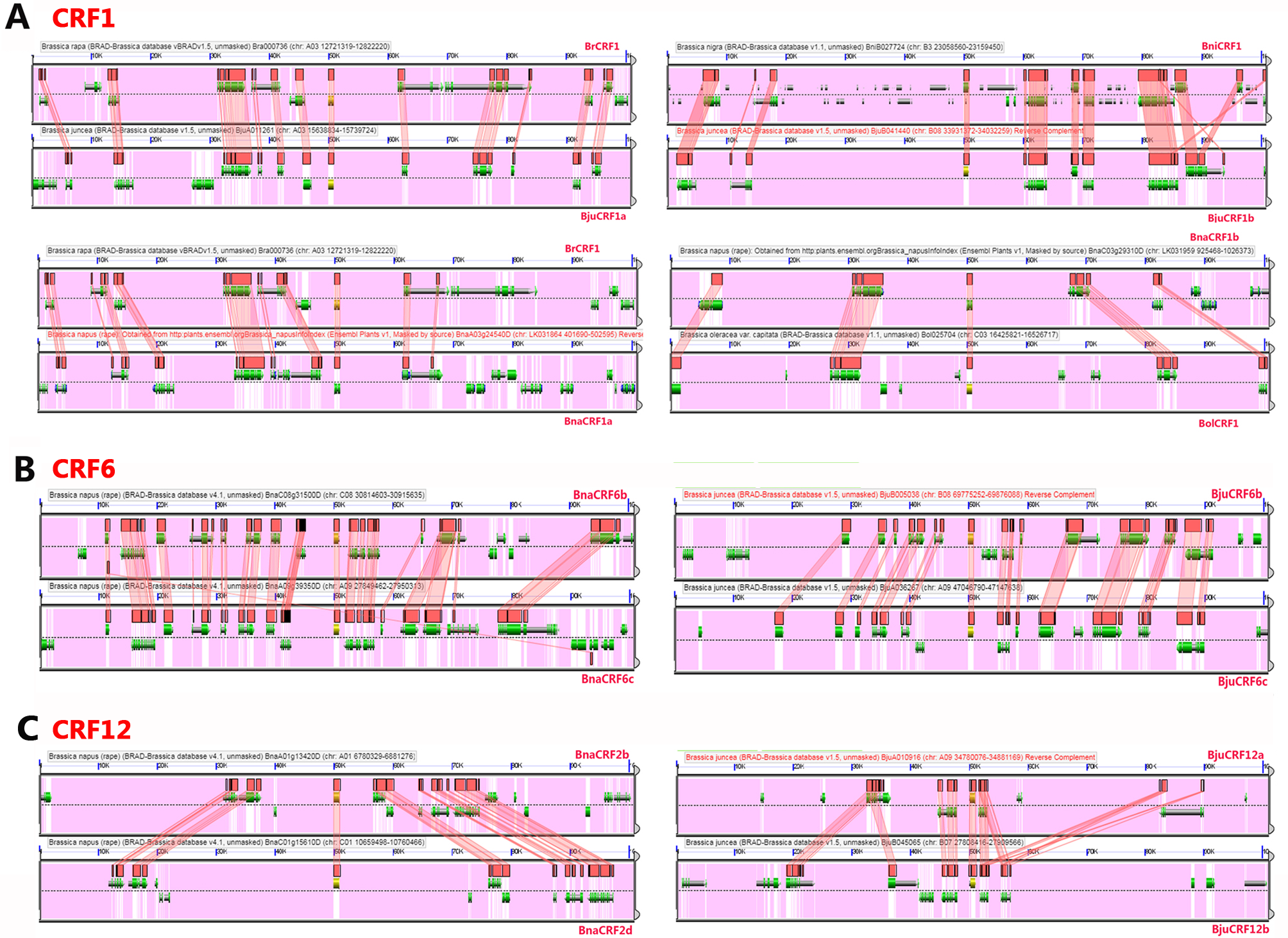

Supplement: Supplementary file 4 — Figure S2. Synteny analysis of CRFs among Brassica species. (A) Alignment between genes in diploid and in allotetraploid. (B) Alignment between similar genes in allotetraploid. Gene models are colored in grey (gene) and green (CDS). Red blocks indicate the high-scoring segment pair (HSP), and pink links show the connectors between HSP.The figure was formed on GEvo (https://genomevolution.org/CoGe/GEvo.pl). (TIF 8633 kb) [file 12864_2018_5114_MOESM4_ESM.tif]

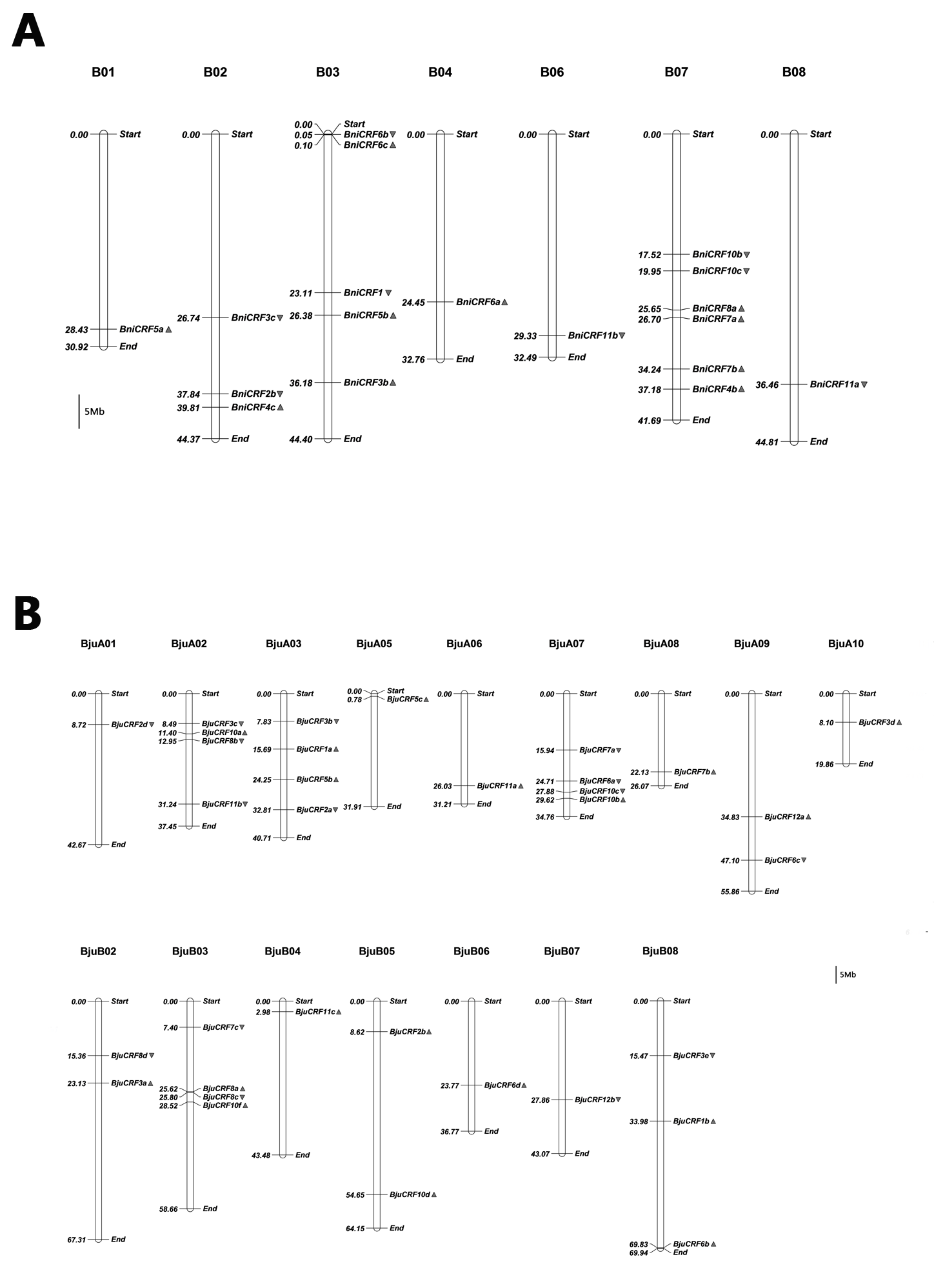

Supplement: Supplementary file 6 — Figure S3. Chromosomal mapping of CRFs in B. nigra and B. juncea. The CRFs in B. nigra (A) and B. juncea (B) are shown except those located on the scaffolds. The locations were shown on the left of the chromosomes, whereas the gene names were on the right. The arrows next to gene names show the direction of transcription. The bar indicates the size of 5 Mb. (TIF 9375 kb) [file 12864_2018_5114_MOESM6_ESM.tif]

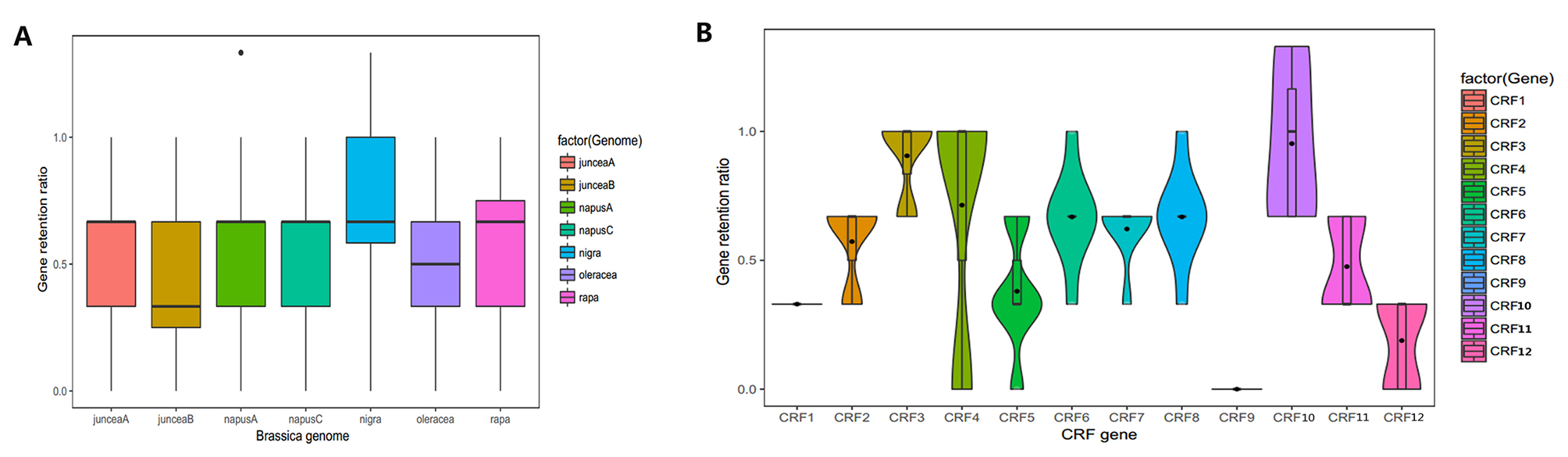

Supplement: Supplementary file 7 — Figure S4. Box-plots of gene retention ratio. Statistics by genomes (A) and by gene names (B) are shown. The Violin plots indicate the number of the same value. (TIF 3464 kb) [file 12864_2018_5114_MOESM7_ESM.tif]

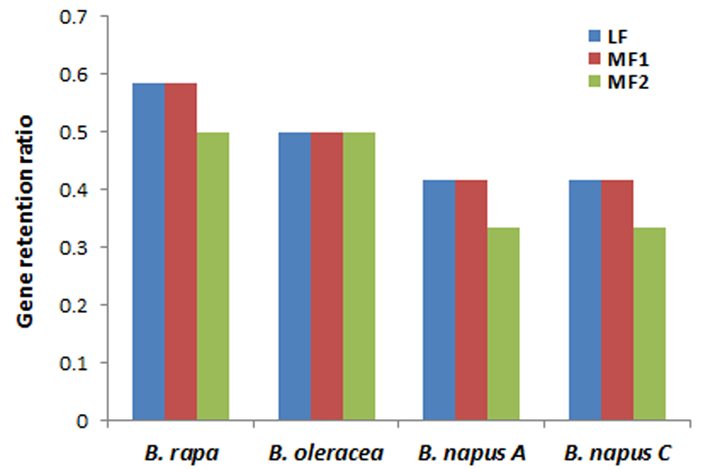

Supplement: Supplementary file 8 — Figure S5. Gene retention ratios of CRFs in the three sub-genomes of B. rapa, B. oleracea, and B. napus. (TIF 1003 kb) [file 12864_2018_5114_MOESM8_ESM.tif]
