# Supplementary material for: Comparative analysis of cytokinin response factors in Brassica diploids and amphidiploids and insights into the evolution of Brassica species
Source: BMC Genomics. 2018 Oct 3;19:728. doi: 10.1186/s12864-018-5114-y (PMC6171139; doi:10.1186/s12864-018-5114-y)
Supplement: Supplementary file 5 — Table S3. List of origin genes in B. rapa, B. nigra and amphidiploid genes in B. napus, B. juncea. High-similarity genes are marked in bold. (DOC 58 kb) [file 12864_2018_5114_MOESM5_ESM.doc]

**Table S3 List of origin genes in *B. rapa,*  *B. nigra* and amphidiploid genes in *B. napus, B. juncea***

| **Origin gene** | **Amphidiploid gene** | |
| --- | --- | --- |
| ***B. rapa*** | ***B. napus*** | ***B. juncea*** |
| *BrCRF1* | *BnaCRF1a* | *BjuCRF1a* |
| *BrCRF2a* | *BnaCRF2a* | *BjuCRF2a* |
| *BrCRF2b* | ***BnaCRF2b***  ***BnaCRF2d*** | *BjuCRF2d* |
| *BrCRF3a* |  | *BjuCRF3d* |
| *BrCRF3b* | *BnaCRF3b* | *BjuCRF3b* |
| *BrCRF3c* | *BnaCRF3c* | *BjuCRF3c* |
| *BrCRF4a* | *BnaCRF4a* |  |
| *BrCRF4b* | *BnaCRF4b* |  |
| *BrCRF5* | *BnaCRF5b* | *BjuCRF5c* |
| *BrCRF6* | *BnaCRF6a* | *BjuCRF6a* |
| *BrCRF7a* |  | *BjuCRF7a* |
| *BrCRF7b* | *BnaCRF7d* | *BjuCRF7b* |
| *BrCRF8a* | *BnaCRF8a* | *BjuCRF8a* |
| *BrCRF8b* | *BnaCRF8d* | *BjuCRF8b* |
| *BrCRF10a* | *BnaCRF10a* | *BjuCRF10a* |
| *BrCRF10b* | *BnaCRF10b* | *BjuCRF10b* |
| *BrCRF10c* | *BnaCRF10c* | *BjuCRF10c* |
| *BrCRF11a* | *BnaCRF11a* | *BjuCRF11a* |
| *BrCRF11b* |  | *BjuCRF11b* |
| *BrCRF12* | *BnaCRF12* | ***BjuCRF12a***  ***BjuCRF12b*** |

| **Origin gene** | **Amphidiploid gene** |
| --- | --- |
| ***B. nigra*** | ***B. juncea*** |
| *BniCRF1* | *BjuCRF1b* |
| *BniCRF2a* | *BjuCRF2c* |
| *BniCRF2b* | *BjuCRF2b* |
| *BniCRF3a* | *BjuCRF3a* |
| *BniCRF3b* | *BjuCRF3e* |
| *BniCRF3c* | *BjuCRF3f* |
| *BniCRF5a* | *BjuCRF5a* |
| *BniCRF6b* | ***BjuCRF6b***  ***BjuCRF6c*** |
| *BniCRF6a* | *BjuCRF6d* |
| *BniCRF7b* | *BjuCRF7c* |
| *BniCRF8a* | *BjuCRF8c* |
| *BniCRF8b* | *BjuCRF8d* |
| *BniCRF10ad* | *BjuCRF10d* |
| *BniCRF10b* | *BjuCRF10e* |
| *BniCRF10c* | *BjuCRF10f* |
| *BniCRF11b* | *BjuCRF11c* |

**Note**: High-similarity genes are marked in bold.
