# Supplementary material for: Comparative analysis of cytokinin response factors in Brassica diploids and amphidiploids and insights into the evolution of Brassica species
Source: BMC Genomics. 2018 Oct 3;19:728. doi: 10.1186/s12864-018-5114-y (PMC6171139; doi:10.1186/s12864-018-5114-y)

**Figure S6** ***Ka/Ks* values of CRF orthologous gene pairs of** ***Br-At, Bol-At* and *Bna-At* over a sliding window of 20 codons.** The x-axis indicates the starting codon of sliding window. The y-axis shows the *Ka/Ks* values.

CRF1


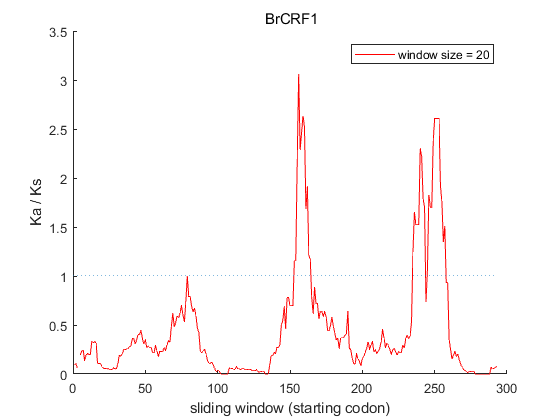

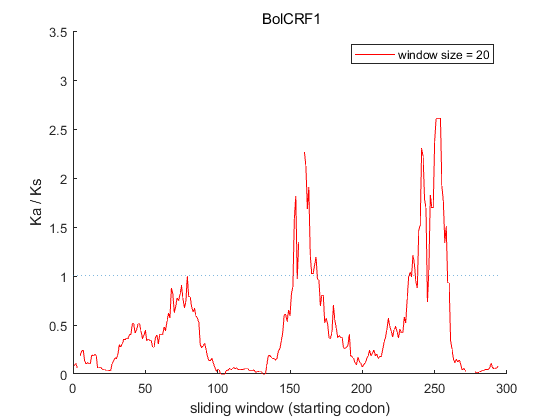

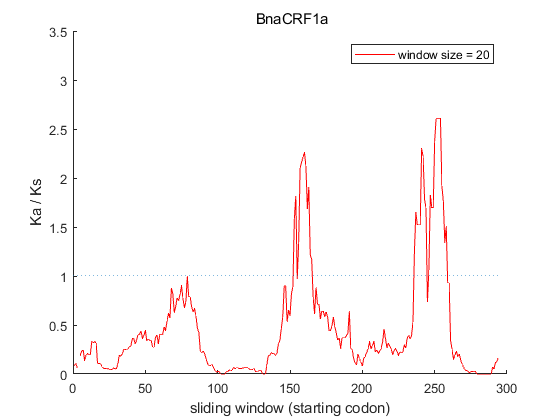

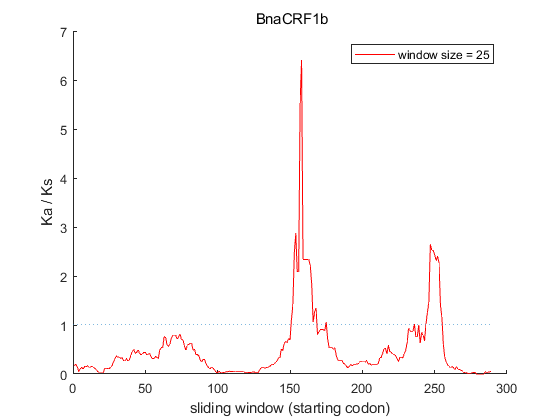


CRF2


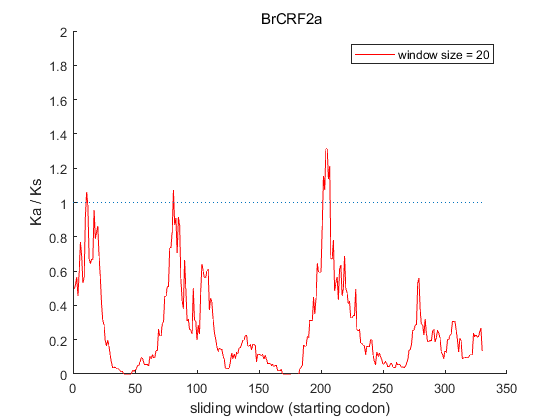

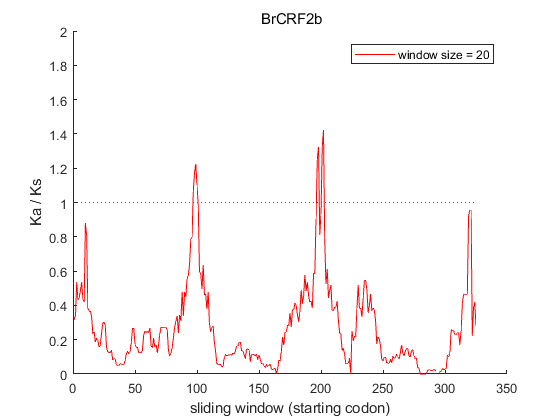


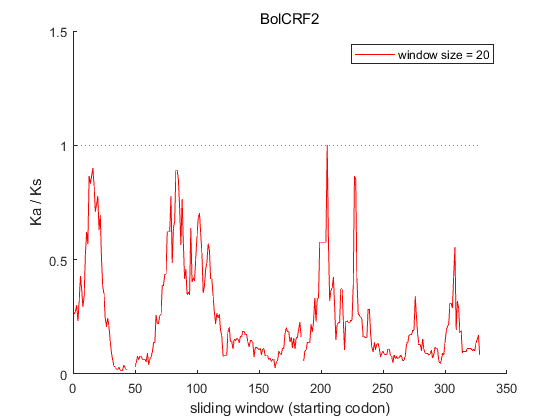

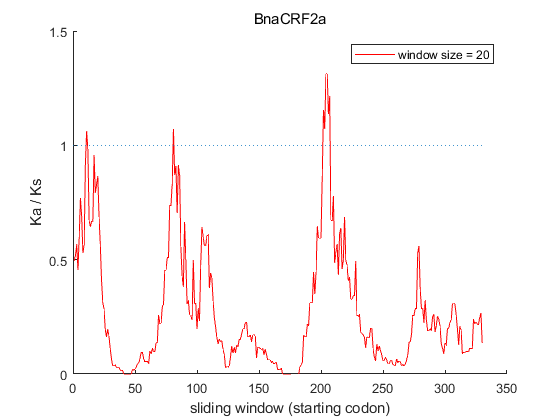


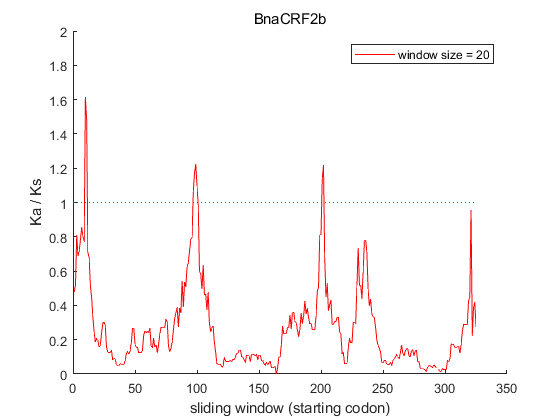

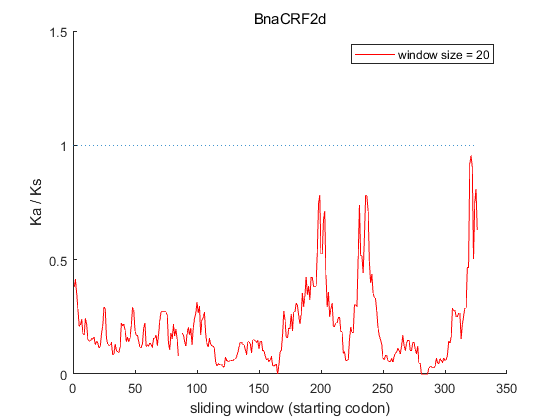


CRF3


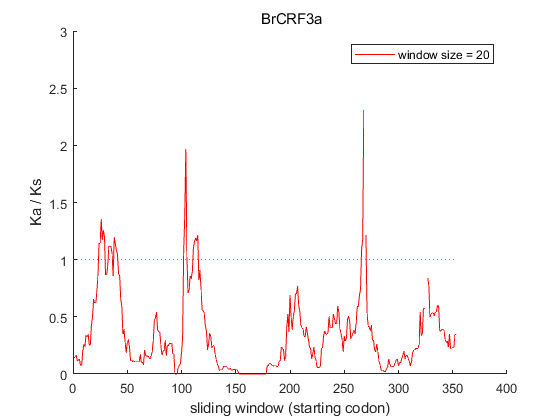

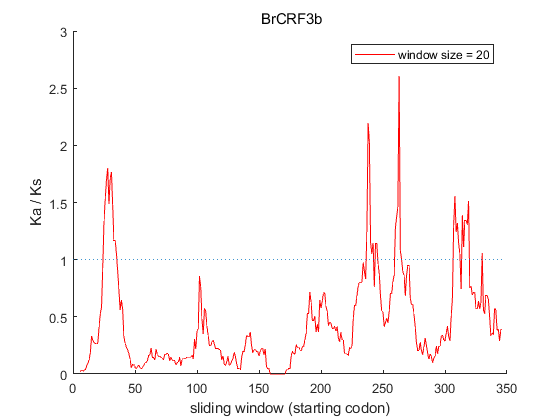

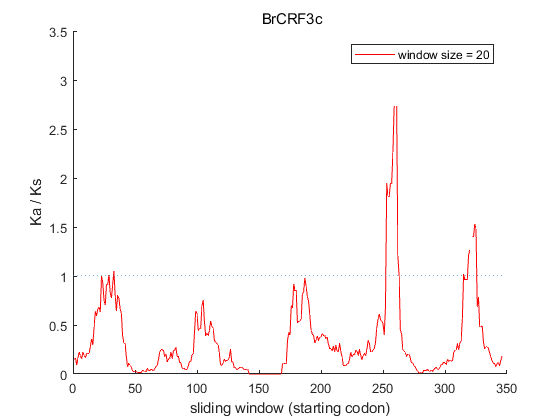

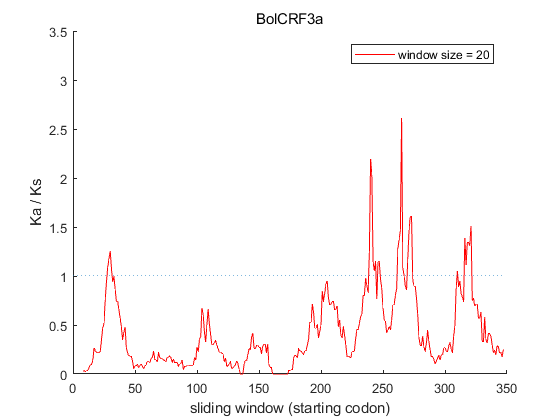


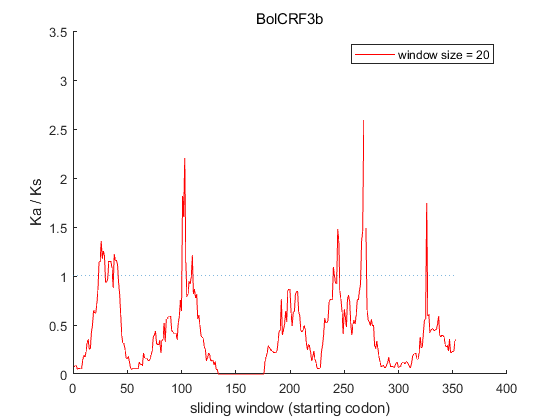

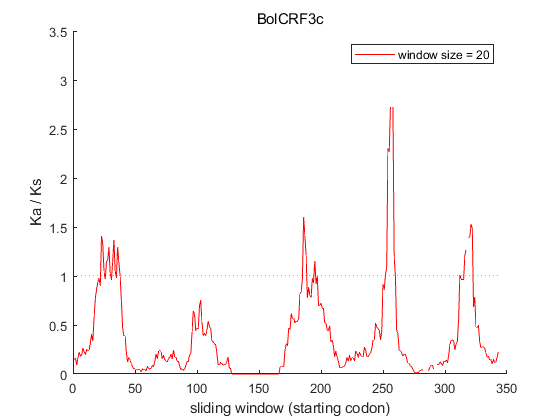

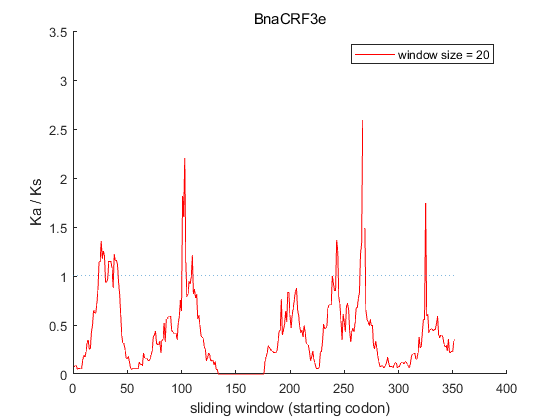

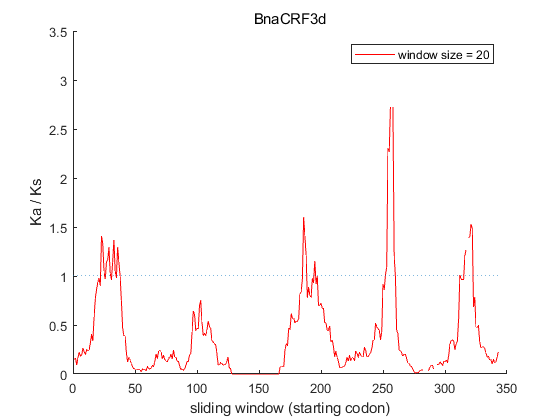

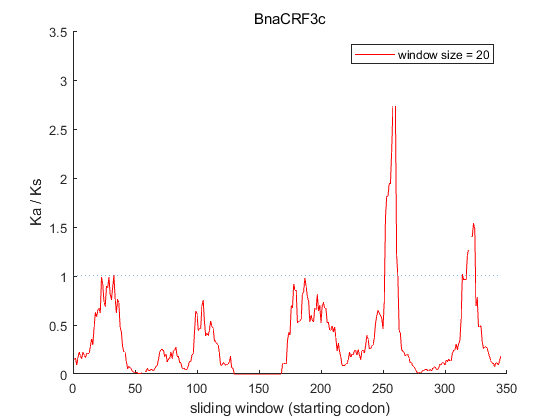

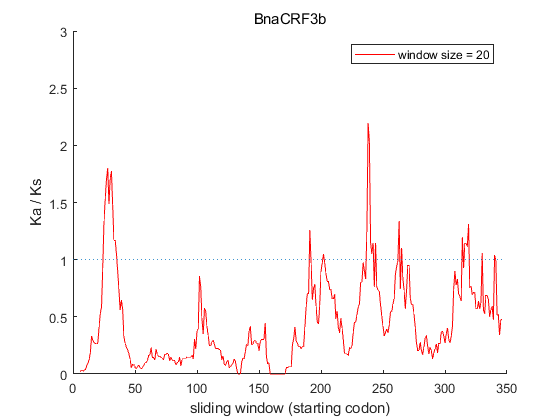

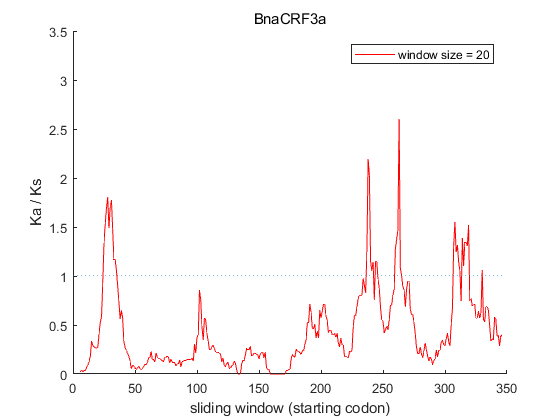


CRF4


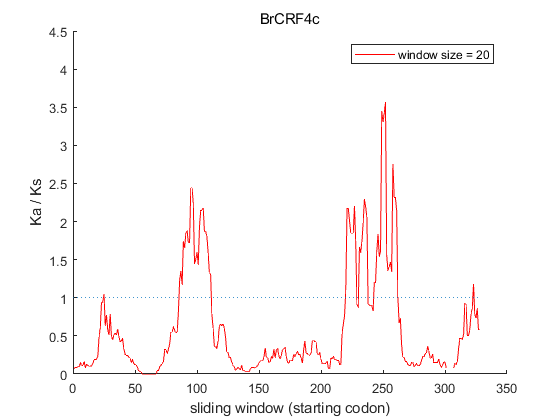

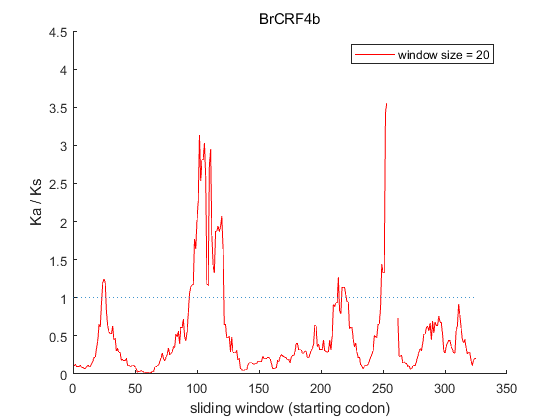

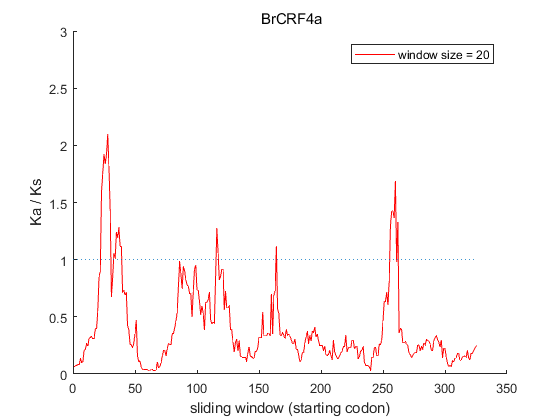

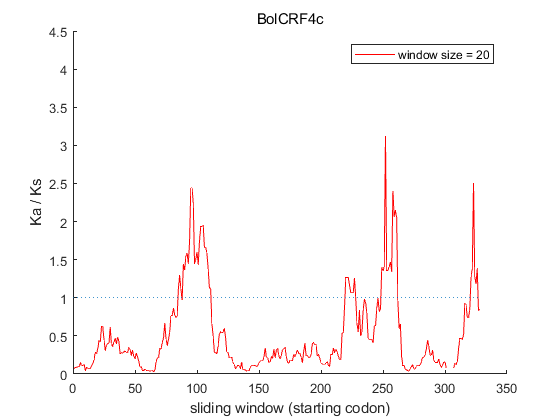

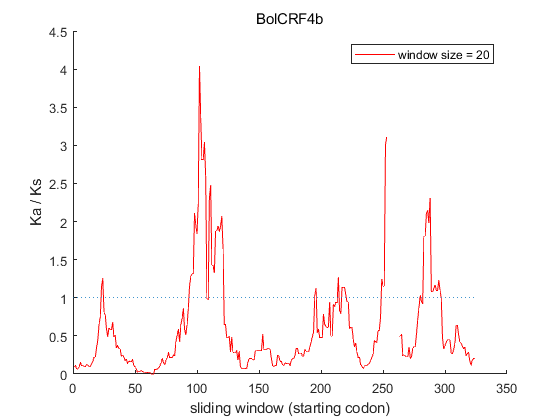

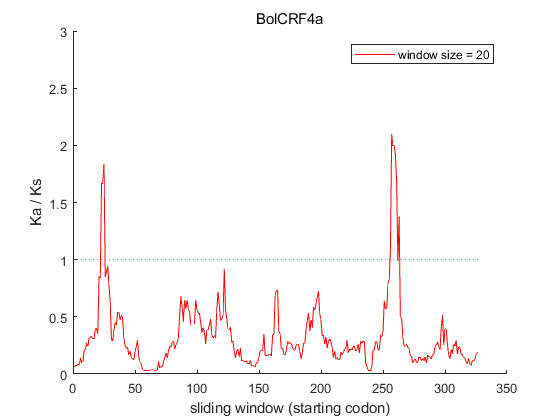

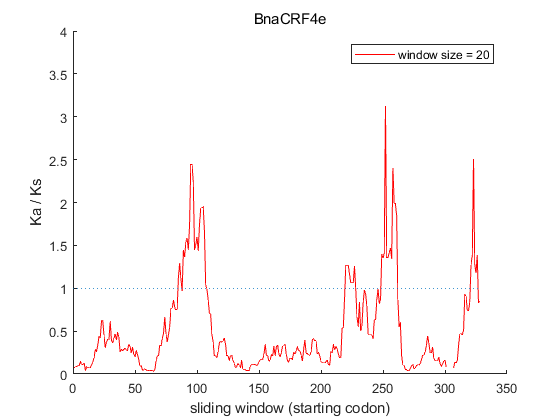

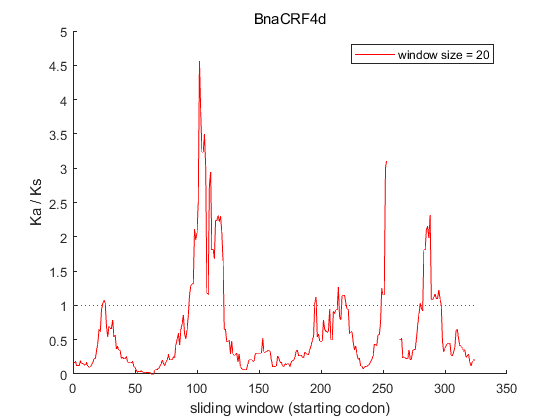

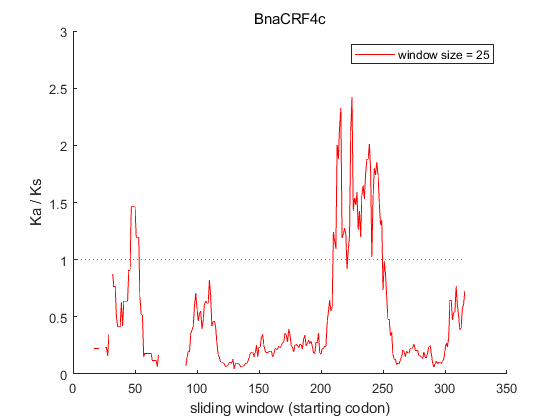

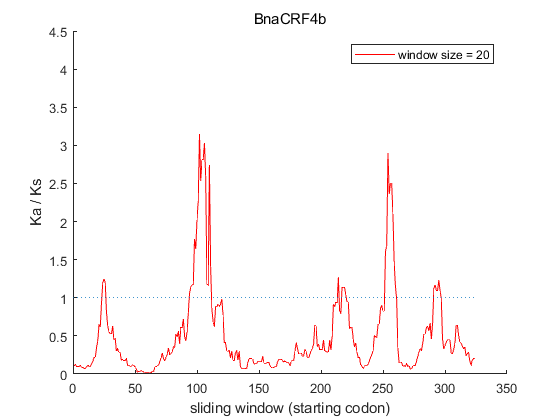

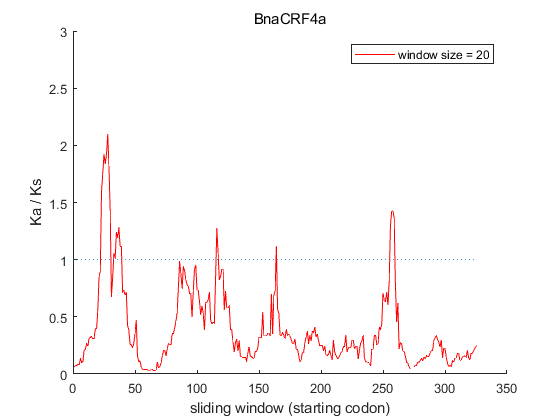


CRF5


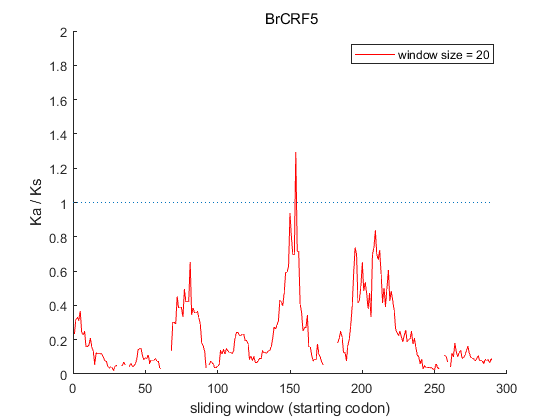

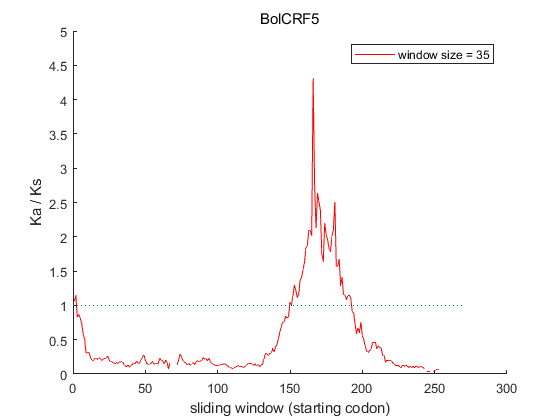

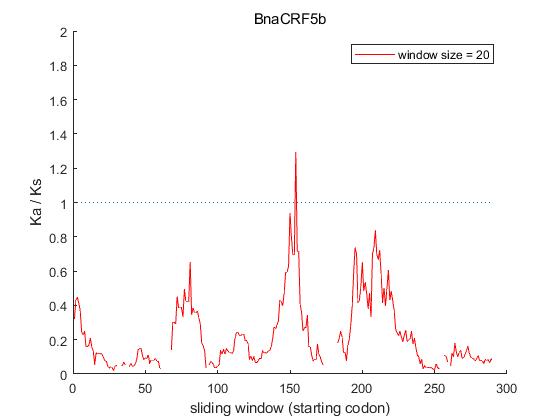

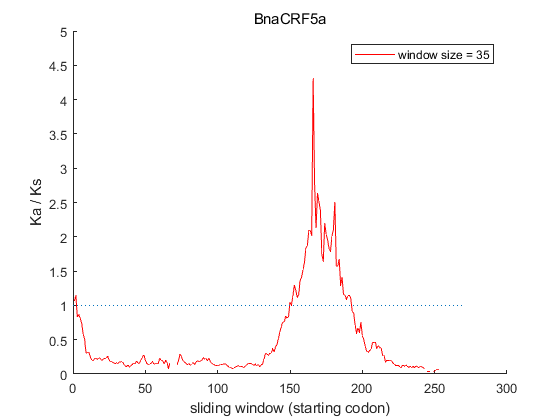


CRF6


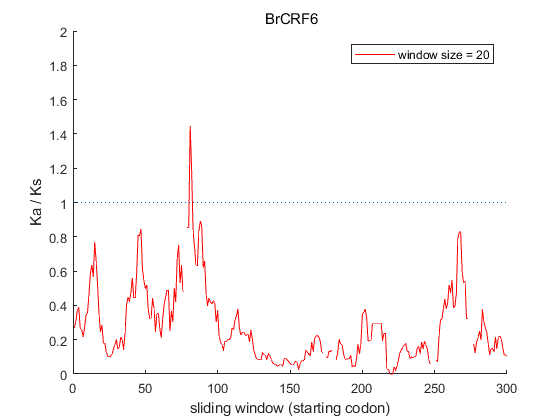

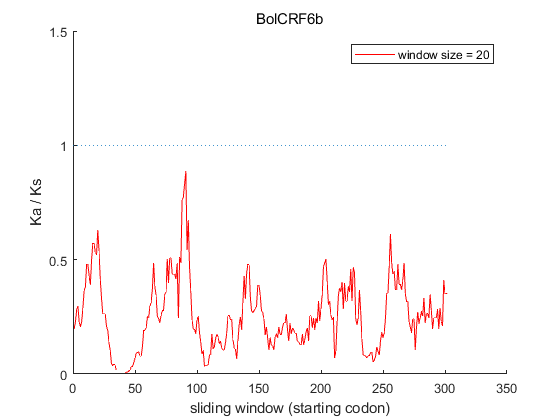

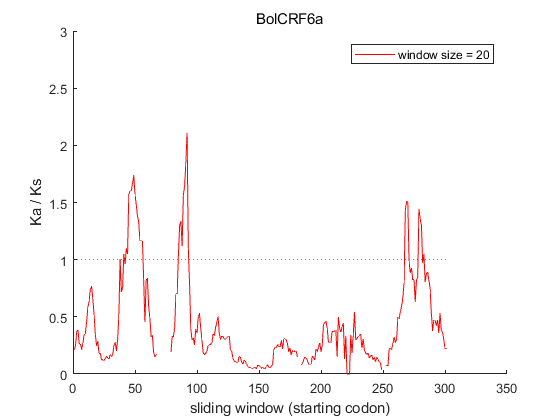

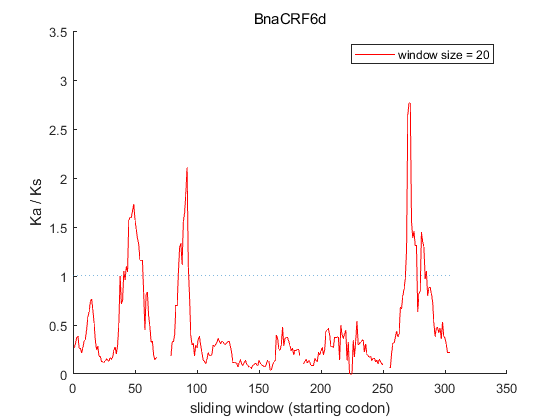

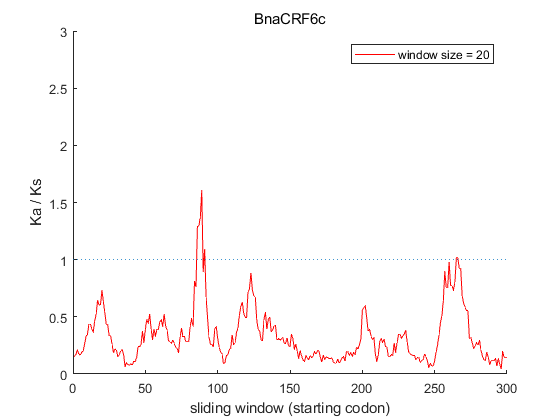

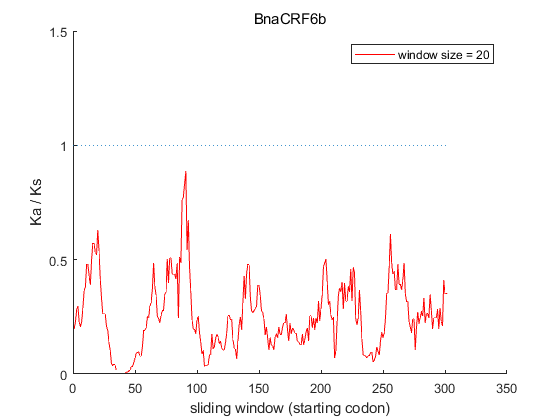

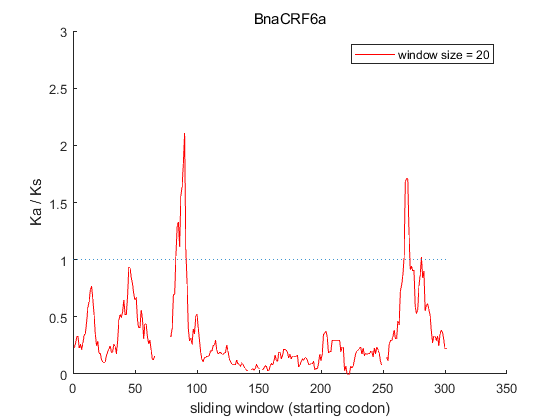


CRF7


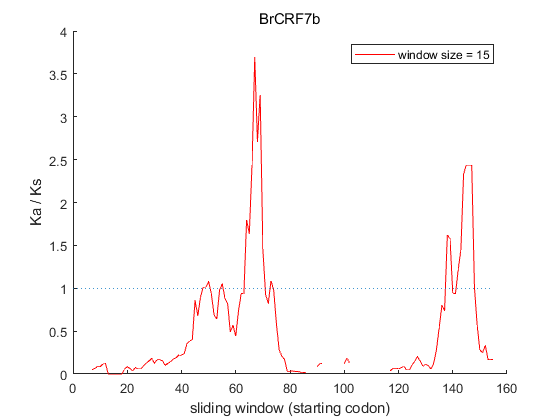

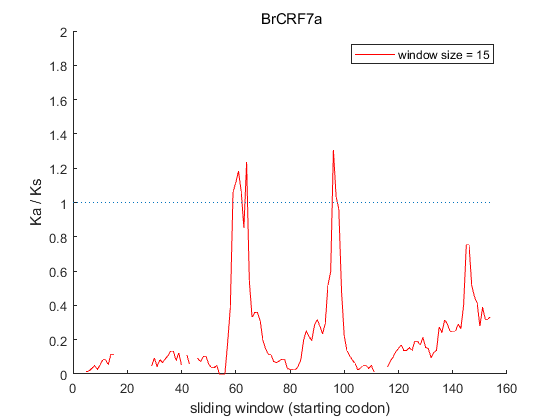

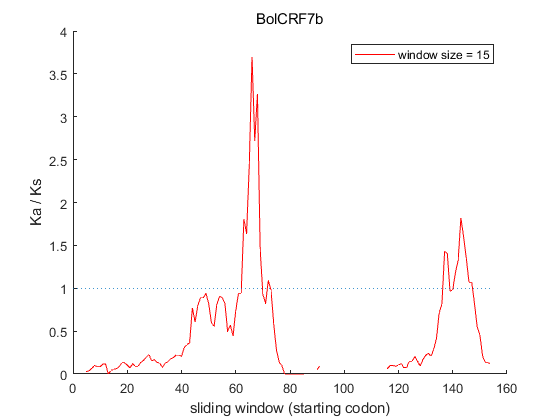

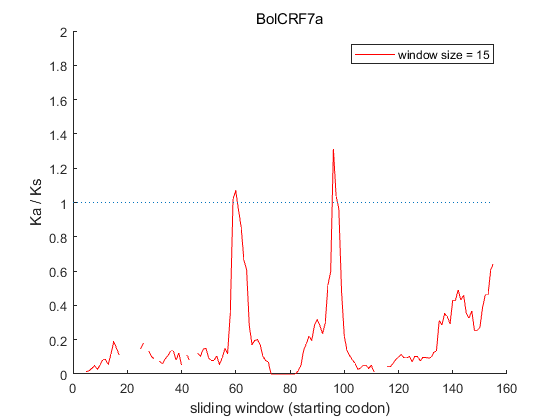


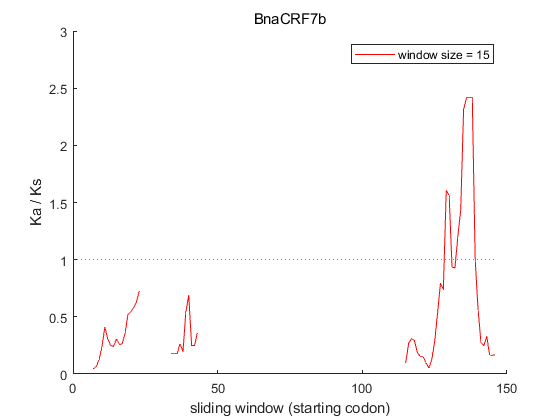

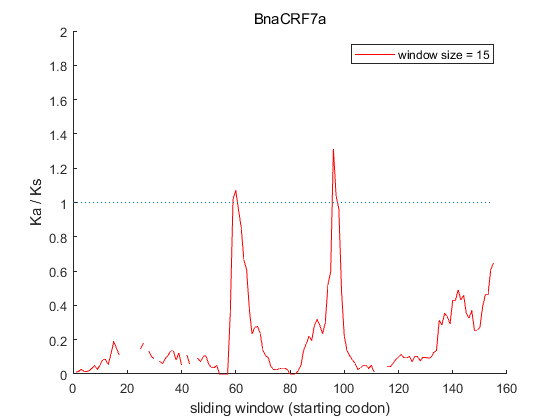


CRF8


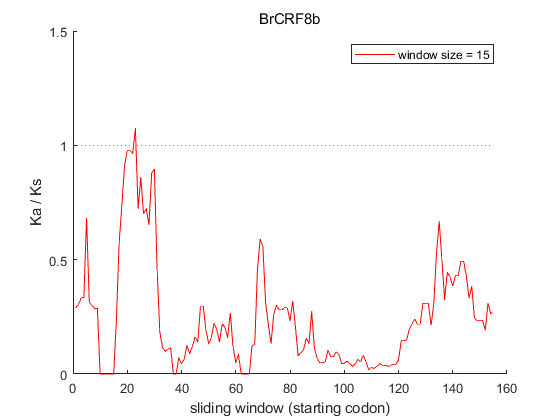

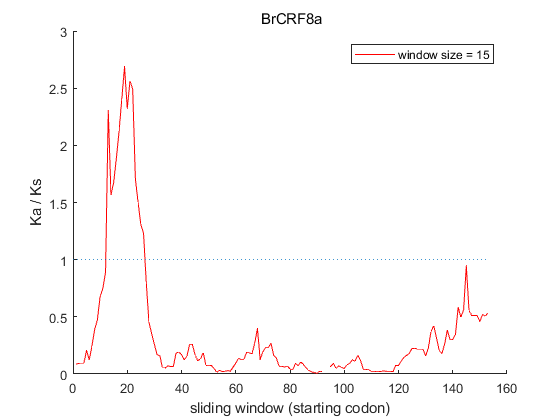


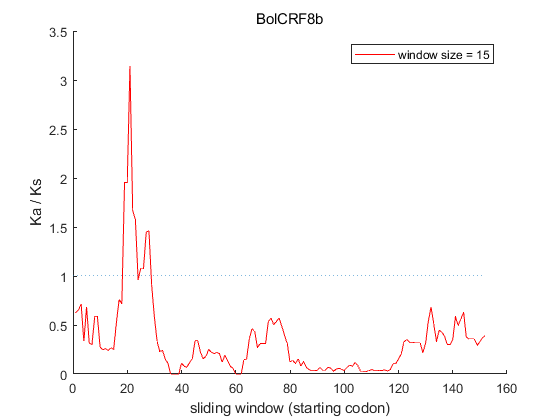

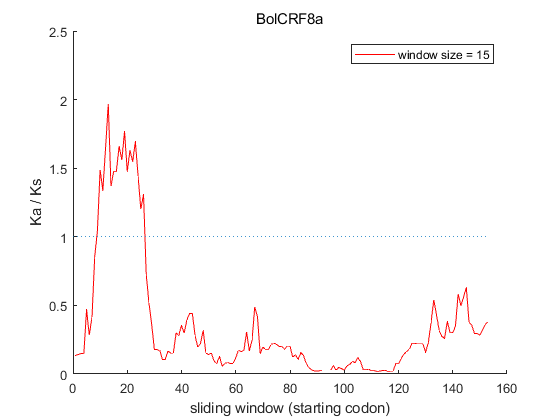

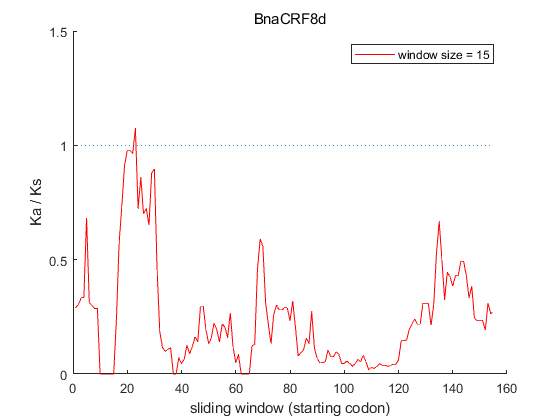

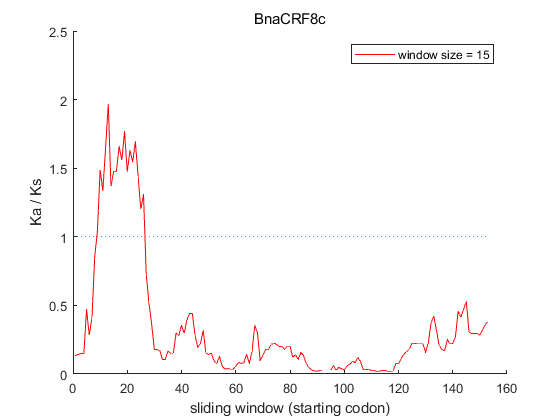


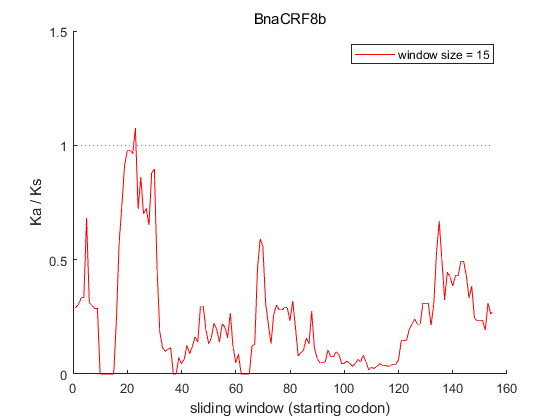

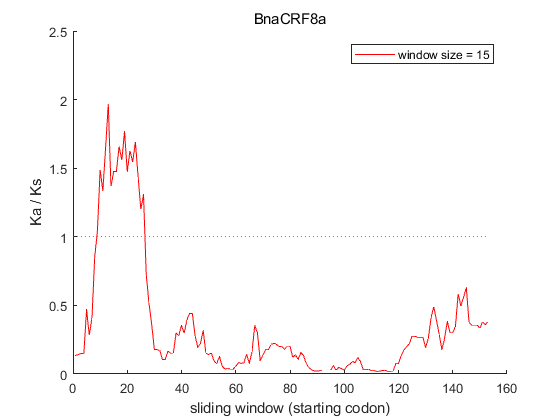


CRF10


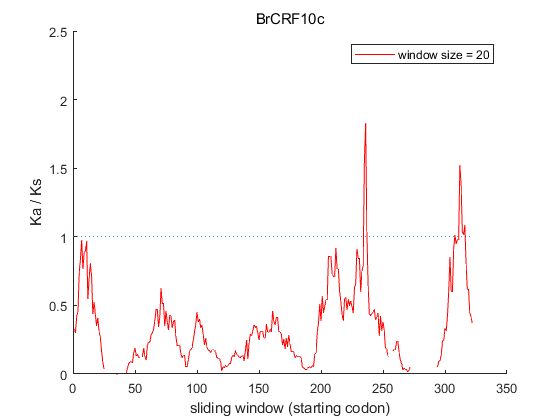

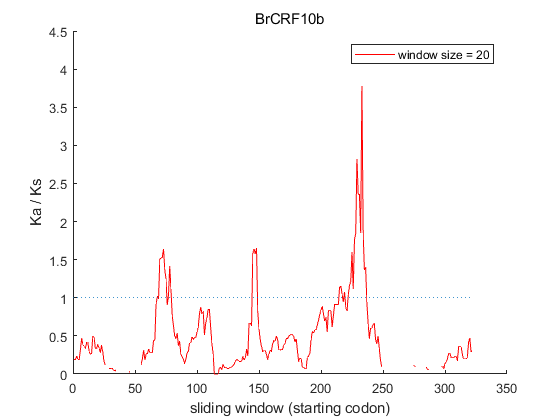


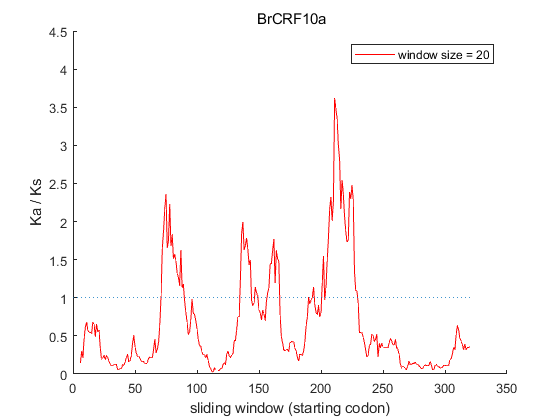

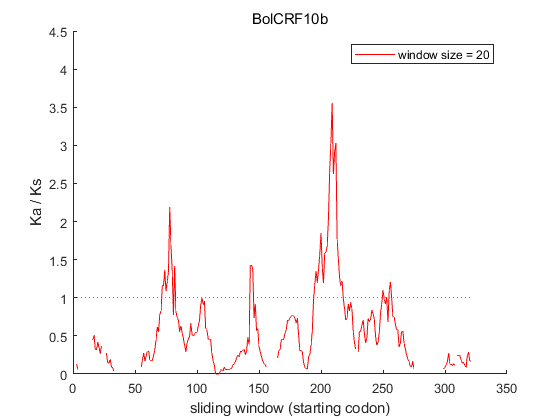

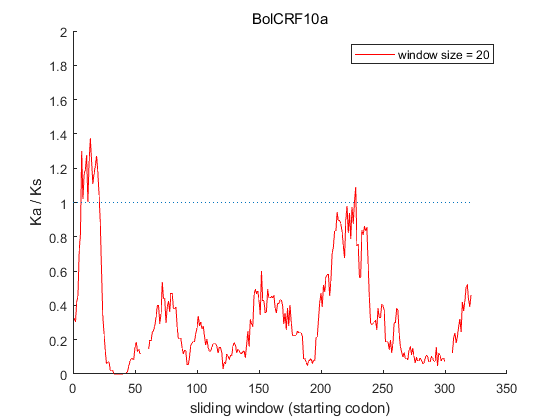

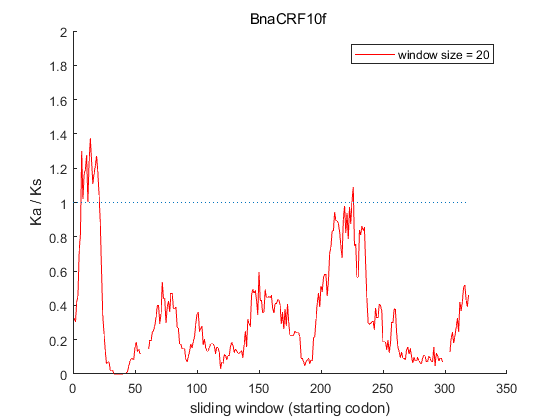

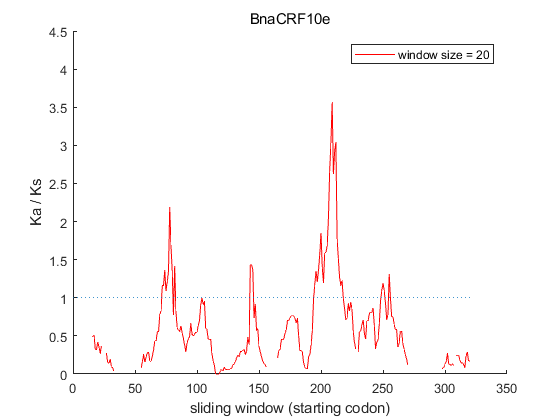

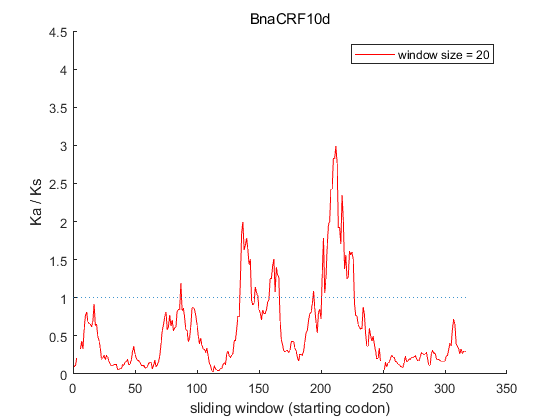

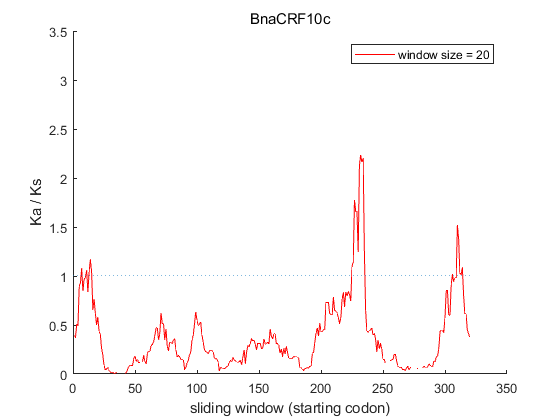

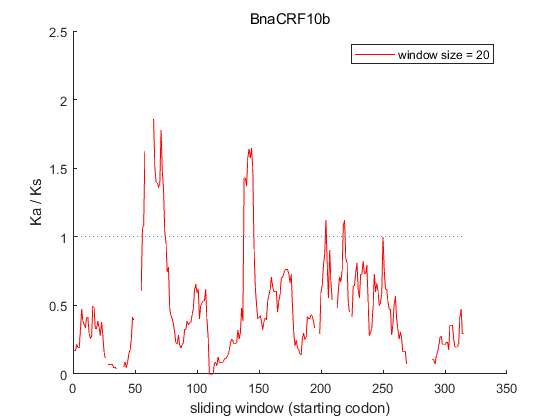


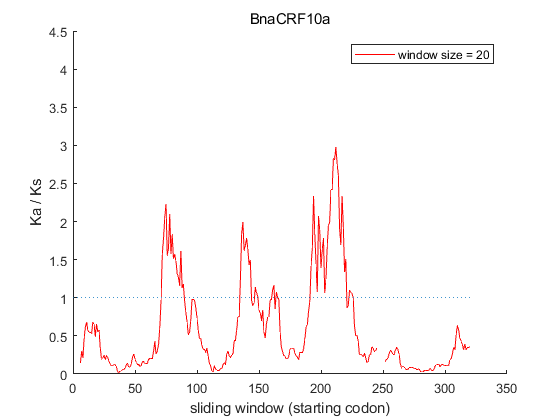


CRF11


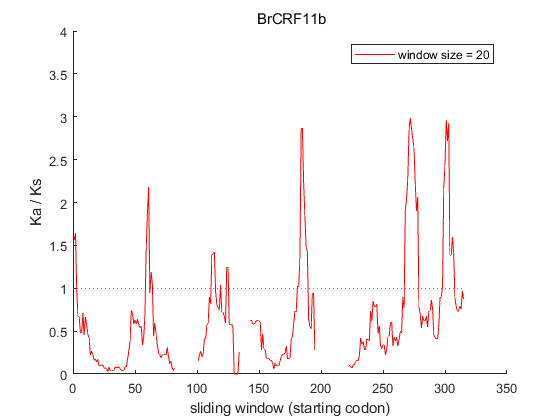

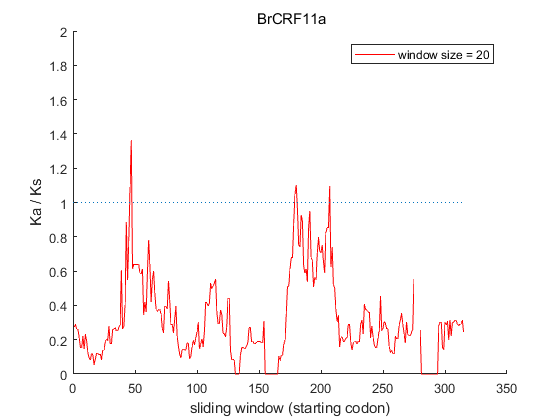


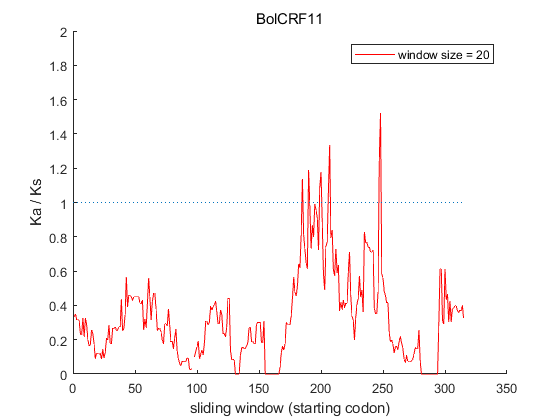

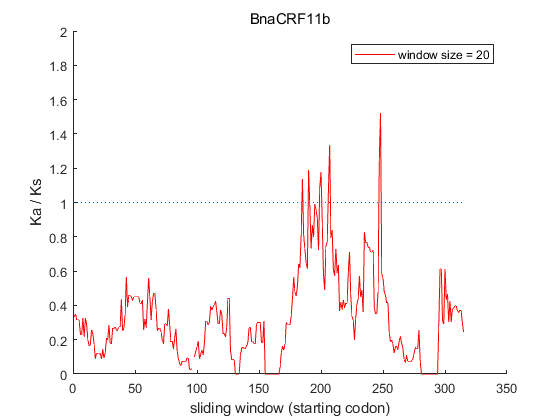

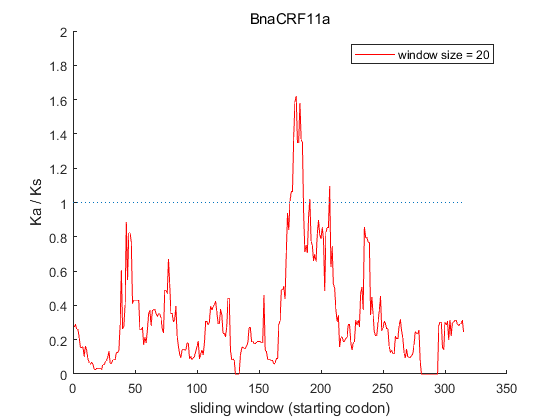


CRF12


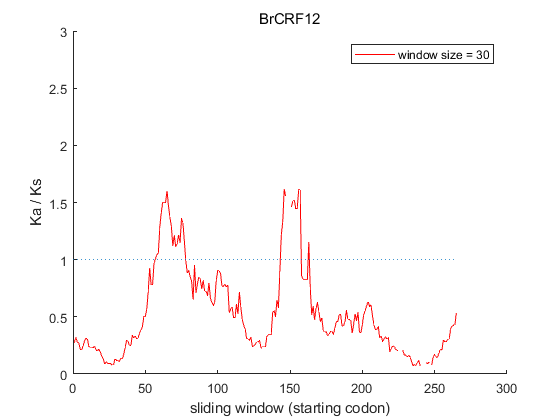

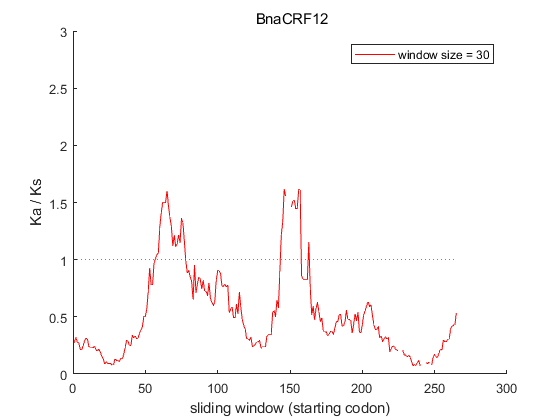

Supplement: Supplementary file 9 — Figure S6. Ka/Ks values of CRF orthologous gene pairs of Br-At, Bol-At and Bna-At over a sliding window of 20 codons. The x-axis indicates the starting codon of sliding window. The y-axis shows the Ka/Ks values. (DOC 1218 kb) [file 12864_2018_5114_MOESM9_ESM.doc]
